# The Malaria Vaccine Programme Evaluation in Kenya: results of a baseline household survey prior to the introduction of the RTS,S/AS01 vaccine

**DOI:** 10.1186/s12936-026-05911-2

**Published:** 2026-04-21

**Authors:** Nelli Westercamp, Isabella Nyangau, Titus K. Kwambai, Brian Seda, Dorcas Akach, Irine Okanda, Elizabeth Marube, Perez Siambe, Eunice Radiro, Florence Wafula, Bellah Ondiegi, Monica P. Shah, Victoria Seffren, Kerryn A. Moore, Ari Fogelson, Paul Milligan, Simon Kariuki, Aaron M. Samuels

**Affiliations:** 1 Malaria Branch, U.S. Centers for Disease Control and Prevention (CDC), 1600 Clifton St MS 16-4, Atlanta, GA 30329 USA; 2 Centre for Global Health, Kenya Medical Research Institute (KEMRI), Kisumu, Kenya; 3 Malaria Branch, U.S. Centers for Disease Control and Prevention (CDC), Kisumu, Kenya; 4 London School of Hygiene and Tropical Medicine, London, UK

**Keywords:** Malaria vaccine, RTS,S/AS01E, RTS,S/AS01E evaluation, Malaria vaccine implementation programme, Malaria vaccine programme evaluation, MVPE, MVIP, Malaria, Vaccine, Implementation research, Vaccine impact, Vaccine safety, Africa, High transmission

## Abstract

**Background:**

In September 2019, Kenya began pilot introduction of the RTS,S/AS01_E_ (RTS,S) malaria vaccine through the World Health Organization (WHO)-coordinated Malaria Vaccine Implementation Programme (MVIP). The pilot, conducted in 46 sub-counties in western Kenya with moderate-to-high perennial malaria transmission, aimed to assess feasibility of delivering the 4-dose schedule through routine immunization services, vaccine safety, and impact on mortality and severe malaria admissions. This paper presents findings from the baseline household survey conducted prior to vaccine introduction to characterize malaria burden, coverage of malaria control interventions, health-seeking behaviors, immunization coverage, and caregiver perceptions of RTS,S.

**Methods:**

A cross-sectional, population-representative household survey was conducted from July to October 2019 in all 46 MVIP sub-counties. Using two-stage cluster sampling, 4065 households were enrolled, including 4948 children aged 5–48 months. Structured questionnaires captured data on insecticide-treated net (ITN) ownership and use, vaccination status, recent febrile illness and care-seeking, and vaccine acceptability. Malaria rapid diagnostic tests were performed to estimate malaria prevalence. Analyses accounted for cluster design and sampling weights.

**Results:**

Overall *P. falciparum* malaria prevalence was 22% (95% CI 19–26), with substantial sub-county variation (1–71%). Prevalence was higher in rural areas, among older children, in lower wealth households, and in areas randomized to vaccine introduction vs. comparison areas, indicating baseline imbalance between the arms. ITN ownership and use were high (93% and 87%). Thirty-eight percent of children had fever in the prior two weeks; 70% sought care, 39% of whom received antimalarials, nearly all artemisinin-combined therapies. Availability of home-based vaccination records declined with age (93% among 5–11 months vs. 63% among 36–48 months). Coverage exceeded 85% for first-year-of-life vaccines, but was lower for measles dose 2 (49%). Vitamin A supplementation (46%) and deworming (50%) coverage were also suboptimal. Before introduction, only 36% of caregivers had heard of the malaria vaccine, yet willingness to vaccinate exceeded 98%.

**Conclusions:**

High malaria burden, strong coverage of core interventions, and strong caregiver support provided a favorable context for RTS,S introduction. However, gaps in second-year-of-life services and suboptimal vaccination records retention may challenge delivery and monitoring of the 4th RTS,S dose. These findings establish a benchmark for evaluating RTS,S rollout and integration into routine child health services.

*Trial registration number* NCT03806465.

**Supplementary Information:**

The online version contains supplementary material available at 10.1186/s12936-026-05911-2.

## Background

In 2024, there were an estimated 282 million malaria cases and 610,000 malaria-related deaths worldwide, with the vast majority occurring in young children in sub-Saharan Africa [1]. Significant investments in vector control interventions and improved case management have contributed to marked reductions in malaria burden, particularly between 2000 and 2015 [2, 3]. In Kenya, malaria prevalence has declined by approximately 88% over the past 20 years; however, the eight counties comprising the lake-endemic region in western Kenya remain highly endemic despite a downward trend in transmission nationally [4]. New challenges have emerged, including the spread of vectors that are resistant to insecticides, [5] and the recent threat of waning artemisinin-based combination therapy (ACT) efficacy and artemisinin partial resistance in the region [6, 7]. Given the persistently high perennial malaria transmission in western Kenya new control tools are urgently needed to address these evolving threats.

In 2015, the European Medicines Agency (EMA) reviewed data on efficacy and safety of the RTS,S/AS01_E_ (RTS,S) malaria vaccine based on findings from a multi-centre Phase 3 trial and earlier studies [8], and issued a positive scientific opinion concluding that the vaccine’s benefits outweigh its risks [9]. Following this, WHO recommended large-scale pilot implementation (Malaria Vaccine Implementation Programme, MVIP) of the four-dose RTS,S regimen in children aged ≥ 5 months in regions with moderate-to-high perennial malaria transmission, with Ghana, Malawi, and Kenya selected based on pre-specified criteria as pilot countries. [10] The Malaria Vaccine Programme Evaluation (MVPE) aimed to assess the feasibility of delivering four vaccine doses, particularly since this schedule extends beyond the existing Expanded Programme on Immunization (EPI) contact points, as well as vaccine safety and impact on mortality and on hospital admissions with severe malaria, during routine use [11].

In September 2019, as part of the broader MVIP, Kenya Ministry of Health (MoH) launched a phased pilot introduction of RTS,S in 46 sub-counties in western Kenya through the routine childhood immunization programme. These sub-counties were randomized to either vaccine introduction (implementing) or to serve as comparison areas for later vaccine introduction pending favorable evaluation findings. Alongside vaccine rollout, an independent feasibility evaluation used three large, population-representative household surveys to characterize pilot sites, assess baseline balance across arms, and measure RTS,S coverage and its impact on other vaccines and interventions at 18- (midline) and 30-months (endline) post-introduction. In this paper, we describe the baseline characteristics of the Kenya MVIP site prior to malaria vaccine introduction, based on the results of the baseline survey.

## Methods

**Fig. 1:**
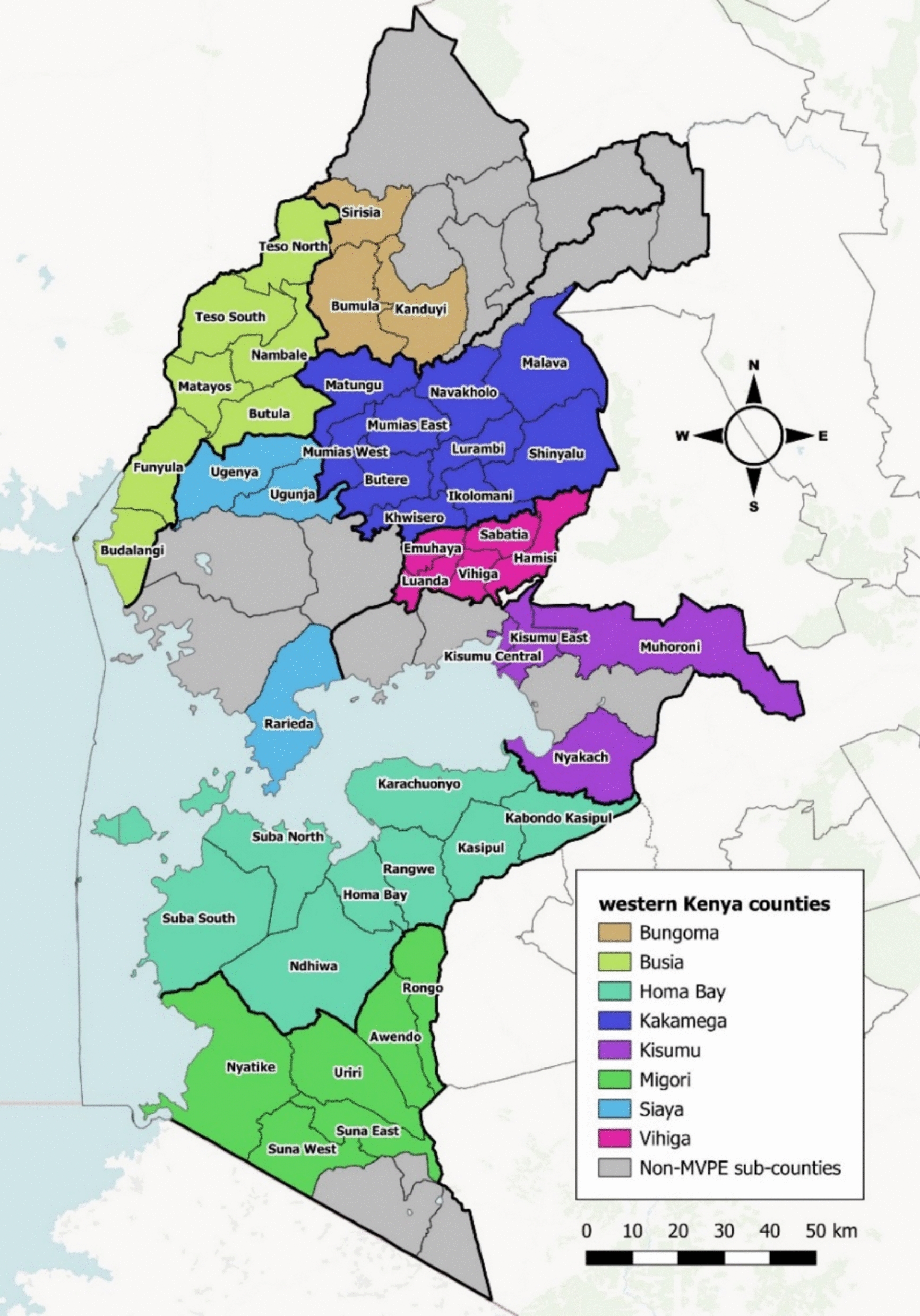
The map of MVIP area in Kenya

A detailed description of the demographic, epidemiologic, health systems context for malaria vaccine introduction [12], and the details of the pilot design and cluster selection have been described elsewhere. [11, 13] The baseline feasibility survey aimed to characterize the MVIP areas and the target population prior to RTS,S introduction, assess balance between study arms, and establish a baseline for evaluating the impact of vaccine introduction on immunization coverage, malaria interventions, child health services, and related outcomes. The survey was conducted in 2019 across all 46 MVIP sub-counties in western Kenya, including 23 RTS,S implementing and 23 comparison sub-counties (Fig. 1), each contributing an annual cohort of approximately 4000 children for the evaluation. These sub-counties span eight counties—Bungoma, Busia, Homa Bay, Kakamega, Kisumu, Migori, Siaya, and Vihiga—that together constitute the lake-endemic zone, a high malaria transmission region with an estimated population 9.8 million. Additional details on methods can be found in supplementary materials.

### Sample size and sampling

A two-stage cluster sampling design was used. In each sub-county, four enumeration areas (EAs) were selected by the Kenya National Bureau of Statistics (KNBS) using probability proportional to estimated population size. Prior to data collection, selected EAs were mapped and households enumerated to establish sampling frames of eligible households. Within each EA, 25 households were randomly selected without replacement using R v3.6.1 (sampler package), targeting 100 households per sub-county. Each household had a known, non-zero probability of selection. All households with children aged 5–48 months were eligible, and all eligible children within selected households were included.

This sample size allowed estimation of coverage indicators with a precision of 10–15% at the sub-county level and precision of ± 2–3% overall (assuming a conservative estimate of 50% coverage, a design effect of 1.5, and high response rate > 95% per cluster). Sampling weights were calculated based on the population size of each EA, non-response adjustment, and the probability of household selection.

### Community sensitization

Community sensitization was conducted in collaboration with national and county health authorities and local administrative leaders. Engagement included meetings with county and sub-county health teams and local leaders within selected EAs to introduce the survey, outline its objectives, and provide an overview of planned activities and timelines.

### Data collection

Data collection was conducted over a 13 week period (July to October 2019), by trained field teams allocated based on local language competencies. The baseline survey methodology was adapted from standard household survey protocols routinely used in Kenya, including the Demographic and Health Surveys (DHS), Malaria Indicator Surveys (MIS), and Multiple Indicator Cluster Surveys (MICS), in consultation with KNBS. A 2-week training was conducted for team supervisors, interviewers, monitors, and quality assurance staff, following the DHS training curriculum.

Questionnaires were designed for maximum comparability with national surveys, developed in English and translated into the local languages (Kiswahili, Dholuo, Maragoli, and Bukusu), and field-tested prior to implementation. Questions assessing vaccine acceptability were drawn from Knowledge, Attitudes, and Practices (KAP) surveys previously implemented for cholera, measles, influenza, and other vaccines, as well as from the MVIP Health Utilization Study [14]. The questionnaires were grouped in four different domains: (1) household information: household location, demographic composition, socio-economic status, and access to malaria prevention; (2) mother/caregiver data: demographics and birth history; (3) child health data: child demographics, immunization history, use of malaria control and other interventions, health seeking behavior for febrile illness, malaria testing and treatment, and nutritional status; (4) vaccine acceptability: perceptions on malaria, acceptability of RTS,S vaccine. Interviews were conducted with mothers or primary caregivers of eligible children. Up to two revisits were made to minimize non-response.

### Informed consent

Written informed consent was obtained from the head of each household and caregivers of eligible children. Consent included permission for the child’s participation in the survey, as well as for malnutrition assessment through mid-upper-arm circumference (MUAC) measurement, malaria testing, treatment, and referral for severe malaria or malnutrition care when needed. Caregivers had an option to respond to the questionnaire but opt out of child’s malaria testing and malnutrition assessment.

All consenting mothers or primary caregivers of children within the eligible age range were interviewed. A separate interview was conducted for each eligible child; therefore, some caregivers were interviewed more than once. In accordance with Kenya's national guidelines for HIV counseling and testing [15], caregivers under the age of 18 were considered mature minors and able to provide consent. Consent forms were translated into the Dholuo, Maragoli, Bukusu, and Kiswahili. Confidentiality was strictly maintained throughout all stages.

### Measurements

#### Immunization history

Immunization history was assessed by requesting to see the home-based immunization record (HBR; Mother and Child Health Booklet in Kenya) to capture vaccinations for each eligible child. If available, dates of all recorded vaccinations were transcribed, and a photo of the HBR was taken. Dose numbering was based on number of doses received, rather than dose timing. When a vaccination was not recorded, the caregiver was asked to recall whether the child received the vaccine. If the HBR was not available, caregivers were asked to recall all vaccinations and number of doses received. An MoH-developed job aid (Supplementary materials, Figure S1) showing different vaccines by age, as well as standardized verbal descriptions of the vaccine administration mode, site, and timing were used to facilitate the caregiver recall. To assess the reliability of caregiver recall, mothers were asked to verbally recall their child’s vaccination history even if the HBR was available.

### Nutritional assessment

Nutritional status was assessed using a color-coded MUAC tape to measure the circumference of the left upper arm at its midpoint. Malnutrition status was defined per standard WHO categories as “at risk” if MUAC was 12.5 cm to < 13.5 cm, moderate acute malnutrition MUAC 11.5cm to < 12.5 cm, and severe acute malnutrition MUAC < 11.5 cm. Due to low numbers of children with acute malnutrition, malnutrition levels were combined into a “risk or presence of malnutrition” variable for MUAC < 13.5 cm for some analyses. Children identified as malnourished were referred for care in accordance with the Kenya National Guidelines for Integrated Management of Acute Malnutrition. [16]

### Malaria testing and treatment

Capillary blood samples were obtained by finger prick to test for *Plasmodium* spp. infection using SD Bioline Malaria Ag P.f/Pan RDT (product code 05FK60; Standard Diagnostics, Inc., Republic of Korea). Children who tested positive by either P.f. or Pan band were treated on site in accordance with Kenya’s National Malaria Treatment Guidelines [17]. Children exhibiting signs of severe malaria or serious illness were referred to the nearest health facility and the local CHV was assigned to follow-up.

### Data management and analysis

Data were collected electronically using Open Data Kit (ODK) with built-in validation checks. Questionnaires were geo-coded and time-stamped to support quality assurance, including tracking of interview duration and verification of field activity locations. Encrypted data were transferred to a secure cloud server with local backups, with ongoing quality control conducted during and after data collection.

All analyses were weighted and accounted for clustering. Descriptive statistics were generated using the survey analysis procedures in SAS v9.4 and Stata v18 estimating immunization coverage, health-seeking behavior, intervention uptake, and other key indicators, and were unadjusted for other indicators. Analyses were stratified by sex, residence (urban/rural), RTS,S implementing and comparison area, socio-economic-status (SES), and malaria prevalence in three groups defined by the tertiles. SES categories, based on tertiles, were derived using principal component analysis of household assets. Following standard WHO EPI coverage assessment methodology, coverage of vaccines scheduled during the first-year-of-life was estimated among children aged 12–23 months, while coverage for vaccines scheduled during the second year of life was estimated among children aged 24–35 months. For the RTS,S vaccine specifically, we applied additional age strata relevant to RTS,S vaccine coverage assessment to facilitate comparability across various indicators with midline and endline surveys: age stratum relevant to RTS,S doses 1–3 was 12–23 months, consistent with other first-year-of-life vaccines; age stratum relevant to RTS,S dose 4 was 30–41 months, reflecting its administration at 24 months with a 6-month interval before evaluation. Additionally, vaccine coverage was calculated based on availability of vaccination records in two ways: 1) by HBR or caregiver recall, if HBR was not available (primary); and 2) by HBR alone (secondary). The analysis evaluating the agreement between the caregiver recall and HBR was carried out on a subset of children for whom both HBR and recall were provided. Agreement between recall and HBR was assessed using percent agreement, Brennan-Prediger coefficients, and Gwet’s AC1 coefficients. An agreement coefficient > 0.70 was considered indicative of acceptable concordance.

### Ethical considerations

The Kenya country-specific MVPE protocol and associated study materials received ethical approval from the following institutions: the Kenya Medical Research Institute (KEMRI) Scientific and Ethical Review Unit (SERU) (Protocol #: 3771), the U.S. Centers for Disease Control and Prevention (CDC) Institutional Review Board (IRB) (Protocol #: 7184), the Oxford Tropical Research Ethics Committee (OxTREC) (Protocol #: 55–18), and the World Health Organization (WHO) Ethical Review Committee (ID: Kenya RTS,S MVIP). The protocol was also reviewed by the Kenya Pharmacy and Poisons Board.

## Results

### Sample description

The baseline survey was conducted from July 15 to October 21, 2019. We enumerated 26,947 households across 184 EAs, from which 5,823 households were randomly selected for enrollment. Of these, 4,103 households met eligibility criteria. Among ineligible households, 69% did not have children of eligible age likely due to population mobility and children aging out in the two-month period between enumeration and survey start; 14% had no caregiver present upon multiple revisits; 9% were vacant or destroyed dwellings, and 8% relocated before the start of the survey. Heads of thirty-eight households (0.7%) declined participation (45% due to lack of interest or perceived benefits, 24% due to concerns about study procedures or vaccines, 20% due to religious or cultural restrictions, and 11% due to time constraints), resulting in the enrollment of 4065 households (88% of intended sample size) with 4169 mothers or caregivers of 4948 children aged 5–48 months. Caregivers of 4888 (99%) children consented for malaria testing and MUAC measurement. On average, each participating household had 1.22 children aged 5–48 months (Supplemental Table S1). When weighted, the sample represents an estimated population of 646,115 children.

Demographic characteristics of caregivers and children are summarized in Table 1. The majority of primary caregivers were mothers (93%) aged between 20 and 40 years (80%). Approximately 64% reported having attained education up to the primary level. The child population was evenly distributed by sex, household wealth tertile, and age group. Equal proportions of children (27%) were aged 12–23 and 24–35 months, contributing to first- and second-year vaccination coverage analyses, respectively. The majority of participants (79%) resided in rural areas.

**Table 1:** Demographic characteristics of caregivers and children

Overall	Implementing	Comparison	Total
	n (%)	n (%)	n (%)
Caregivers	2067 (100)	2102 (100)	4169 (100)
Caregiver type			
Mother	1892 (92)	1968 (94)	3860 (93)
Other	175 (8)	134 (6)	309 (7)
Caregiver age			
< 20 years	86 (4)	79 (4)	165 (4)
20–29 years	934 (45)	921 (44)	1855 (45)
30–39 years	731 (35)	778 (37)	1509 (36)
40–49 years	171 (8)	205 (10)	376 (9)
> = 50 years	145 (7)	119 (6)	264 (6)
Caregiver education			
Primary or below	1341 (65)	1332 (63)	2673 (64)
Secondary/"A" level	532 (26)	570 (27)	1102 (26)
College/Middle level	171 (8)	170 (8)	341 (8)
University	23 (1)	30 (1)	53 (1)
Children	2430 (100)	2518 (100)	4948 (100)
Age group			
5–11 months	366 (15)	394 (16)	760 (15)
12–23 months	644 (27)	694 (28)	1338 (27)
24–35 months	665 (27)	652 (26)	1317 (27)
36–48 months	755 (31)	778 (31)	1533 (31)
Sex			
Male	1256 (52)	1279 (51)	2535 (51)
Female	1174 (48)	1239 (49)	2413 (49)
Residence			
Urban	630 (26)	405 (16)	1035 (21)
Rural	1800 (74)	2113 (84)	3913 (79)
Wealth index			
Low	792 (33)	886 (35)	1678 (33)
Medium	879 (36)	827 (33)	1706 (35)
High	759 (31)	805 (32)	1564 (32)

### Malaria prevalence and interventions access and use

**Fig. 2:**
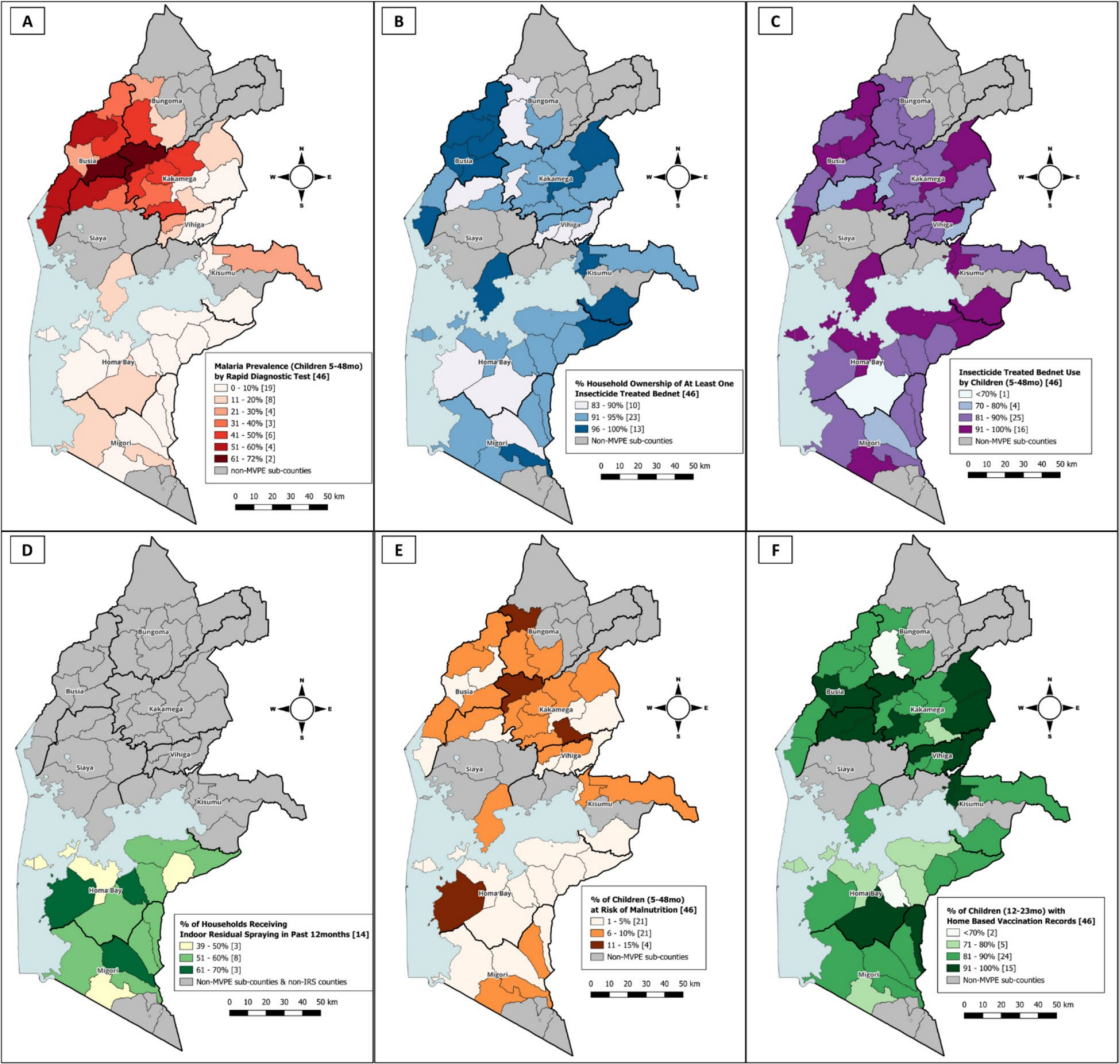
Maps of distribution of survey indicators: **A** – malaria prevalence, **B** – insecticide-treated net (ITN) ownership, **C** – ITN use by children the night before the survey, **D** – indoor residual spraying (IRS) coverage, **E** – malnutrition prevalence, and **F** – home-based record (HBR) coverage. *Scales were tailored to the distribution and meaningful ranges of each indicator*

The overall *Plasmodium falciparum* malaria prevalence among children aged 5–48 months was 22% (95% CI 19–26), with substantial geographic heterogeneity across MVPE sub-counties, ranging from 1 to 71% (Table 2, Fig. 2A; sub-county-specific estimates available in Supplemental Table S2). No significant patterns in prevalence by month of data collection were observed (Supplemental Figure S2). The lowest prevalence was observed in areas implementing indoor residual spraying (IRS; all 8 sub-counties in Homa Bay and 6 sub-counties in Migori counties) and the highest prevalence was observed in some sub-counties within Bungoma, Kakamega, and Busia counties. Malaria prevalence increased with child age, with prevalence ratios (PR) of 1.47 (95%CI 1.17–1.84) among children aged 12–23 months, 1.61 (95%CI 1.25–2.06) among those aged 24–35 months, and 2.04 (95%CI 1.62–2.57) among those aged 36–48 months, compared to children aged 5–11 months. Among age groups relevant for assessing RTS,S malaria vaccine coverage in subsequent feasibility surveys, malaria prevalence was 20% (95% CI: 17–24) in the RTS,S dose 1–3 coverage age group (12–23 months) and 22% (95% CI 18–28) in the age group that would be used to assess RTS,S dose-4 coverage (30–41 months). Considerably higher malaria prevalence was observed in rural compared to urban areas (PR 2.72, 95% CI 1.63–4.54). Estimated malaria prevalence was 1.42 (95%CI 1.12–1.80) times higher in areas randomized to malaria vaccine implementation (26%; 95%CI 23–31) than in comparison areas (19%; 95%CI 16–22). Prevalence was lower among children from medium and high wealth tertiles compared to those in the lowest tertile, among children who slept under an insecticide-treated net (ITN) the night before the survey, and among children residing in IRS sub-counties (Table 2).

**Table 2:** Malaria prevalence in children 5–48 months of age

Background characteristic	Malaria prevalence		Prevalence ratio (PR)
	n/N unweighted	% (95% CI) weighted	
Overall	1144/4888	22 (19, 26)	–
Age group			
5–11 months	113/751	14 (11, 18)	Reference
12–23 months	285/1327	20 (17, 24)	1.47 (1.17, 1.84)*
24–35 months	160/674	22 (19, 26)	1.61 (1.25, 2.06)*
36–48 months	435/1512	28 (25, 32)	2.04 (1.62, 2.57)*
Sex			
Female	547/2376	22 (19, 25)	0.96 (0.85, 1.07)
Male	597/2512	23 (20, 26)	Reference
Residence			
Urban	127/1023	9 (6, 15)	Reference
Rural	1017/3865	26 (23, 29)	2.72 (1.63, 4.54)*
Randomization arm			
Implementing	642/2400	26 (23, 31)	1.42 (1.12, 1.80)*
Comparison	502/2488	19 (16, 22)	Reference
Wealth index (tertile)			
Low	514/1664	30 (26, 34)	Reference
Medium	424/1690	25 (21, 29)	0.83 (0.71, 0.97)*
High	206/1534	12 (10, 15)	0.42 (0.34, 0.51)*
Insecticide-treated net (ITN) use			
Slept under ITN last night	940/4265	21 (19, 24)	0.69 (0.57, 0.84)*
Did not sleep under ITN last night	204/623	31 (25, 38)	Reference
Indoor residual spraying (IRS) implementation area			
IRS implementation areas	86/1532	6 (2, 10)	0.23 (0.12, 0.45)*
Non-IRS areas	1058/3356	30 (25, 34)	Reference

*p < 0.05

Overall, 93% (95% CI: 92–94) of households owned at least one ITN (average 2.12 ITNs per household), with geographic variation across sub-counties ranging from 83 to 100% (Table 3; Fig. 2B; Supplemental Table S2). Reported ITN use the night before the survey among children was 87% (95% CI 85–89), with sub-county-level estimates ranging from 69 to 98% (Table 3; Supplemental Table S2; Fig. 2C). No substantial differences in ITN ownership or use were observed by residence (urban vs. rural), randomization arm, or malaria prevalence strata. However, slightly higher ownership and use were noted among households in the upper wealth groups. IRS had been implemented in 14 sub-counties (5 in the vaccine implementing arm and 9 in the comparison arm) in Homa Bay and Migori counties (Fig. 2D). Among households in IRS-implementing areas, 55% (95% CI 51–59) reported being sprayed within the 12 months preceding the survey, with coverage varying from 39 to 70% across IRS sub-counties (Supplemental Table S2).

**Table 3:** Insecticide-treated net ownership and reported use among children 5–48 months of age the night before the survey

Background characteristic	Number of households	Average number of nets per household	Percent of households with at least one net			Percent of children who slept under a net last night		
	n (un-weighted)	Mean (SE) weighted	n/N (un-weighted)	% (95%CI) weighted	PR (95%CI)	n/N (un-weighted)	% (95%CI) weighted	PR (95%CI)
Overall	4047	2.12 (0.03)	3747/4047	93 (92, 94)		4312/4948	87 (86, 89)	–
Residence								
Urban	853	2.01 (0.06)	788/853	94 (92, 96)	Ref.	917/1035	91 (87, 94)	Reference
Rural	3194	2.14 (0.04)	2959/3194	93 (92, 94)	0.98 (0.96, 1.01)	3395/3913	86 (85, 88)	0.95 (0.91, 1.00)
Randomization arm								
Implementing	1999	2.10 (0.05)	1841/1999	92 (91, 94)	0.98 (0.96, 1.01)	2094/2430	86 (83, 88)	0.96 (0.93, 1.00)
Comparison	2048	2.13 (0.04)	1906/2048	94 (93, 95)	Ref	2218/2518	89 (87, 91)	Reference
Malaria prevalence (tertile)								
Low	1278	2.13 (0.07)	1190/1278	94 (92, 96)	Ref.	1475/1672	90 (88, 92)	Reference
Medium	1406	2.05 (0.05)	1286/1406	92 (90, 94)	0.98 (0.95, 1.02)	1396/1617	85 (82, 89)	0.95 (0.91, 0.99)*
High	1363	2.18 (0.05)	1271/1363	93 (92, 95)	0.99 (0.97, 1.02)	1441/1659	86 (84, 89)	0.96 (0.93, 1.00)
Wealth index (tertile)								
Low	1339	1.83 (0.05)	1191/1339	89 (87, 91)	Ref.	1366/1678	81 (78, 84)	Reference
Medium	1379	2.10 (0.05)	1279/1379	93 (92, 95)	1.05 (1.01, 1.08)*	1484/1706	87 (85, 89)	1.08 (1.03, 1.12)*
High	1329	2.41(0.06)	1277/1329	96 (95, 98)	1.08 (1.06, 1.11)*	1462/1564	94 (93, 95)	1.16 (1.12, 1.21)*

* p < 0.5

### Care-seeking behaviors

Care-seeking behavior for febrile illness among children aged 5–48 months is summarized in Table 4. Thirty-eight percent (95% CI 36–41) of children were reported to have fever in the 2 weeks preceding the survey, and among them, 70% (67–72) sought care. Of those who sought care, 34% (32–37) did so within one day of fever onset, 54% (51–57) were reported being tested for malaria by finger or heel prick, and 39% (36–44) received antimalarial treatment, with 97% of antimalarials being ACTs. There was no difference in care-seeking behavior or care experience by sex, urban or rural residence, randomization arm, or household wealth index. Children residing in areas with the highest malaria prevalence were more likely to report febrile illness, but less likely to seek care; however, those who sought care were more likely to be treated with antimalarials, including ACTs, than children from areas with lower malaria prevalence. In contrast, children from areas with IRS were less likely to report recent fever but more likely to seek care when febrile. Children in the two RTS,S coverage age cohorts (12–23 months for doses 1–3 and 30–41 months for dose-4) had care-seeking behaviors and experiences similar to those of the overall survey sample.

**Table 4:** Care seeking behavior for febrile illness in children

				Among children with fever in past 2 weeks who sought care			
Background characteristic	Number of children with fever in past 2 weeks	Percentage with fever in past 2 weeks	Percentage who sought advice or treatment	Percentage reported testing by finger or heel stick	Percentage who took an antimalarial drug	Percentage treated with artemisinin-based combination therapy (ACT)	Percentage who sought treatment within 1 day of fever
	n unweighted	% (95%CI) weighted	% (95%CI) weighted	% (95%CI) weighted	% (95%CI) weighted	% (95%CI) weighted	% (95%CI) weighted
Overall	1805/4932	38 (36, 41)	70 (67, 72)	54 (51, 57)	39 (36, 44)	38 (36, 41)	34 (32, 37)
Age group							
5–11 months	287/758	40 (36, 44)	77 (71, 83)	55 (47, 64)	29 (23, 38)	31 (25, 38)	35 (30, 42)
12–23 months	508/1333	41 (37, 45)	72 (68, 76)	60 (55, 64)	41 (35, 48)	39 (35, 45)	36 (31, 41)
24–35 months	251/678	38 (33, 43)	71 (65, 78)	49 (43, 56)	40 (33, 48)	36 (31, 43)	37 (31, 45)
36–48 months	525/1528	35 (32, 39)	67 (59, 76)	51 (46, 57)	43 (36, 51)	43 (37, 49)	30 (27, 35)
Gender of child							
Female	832/2403	36 (33, 39)	69 (66, 73)	52 (48, 56)	38 (33, 44)	37 (33, 41)	33 (29, 38)
Male	973/2529	41 (38, 44)	70 (66, 74)	55 (52, 59)	40 (36, 45)	39 (36, 43)	35 (32, 40)
Residence							
Urban	342/1032	35 (28, 43)	70 (63, 77)	55 (48, 62)	35 (29, 43)	33 (27, 40)	38 (33, 45)
Rural	1463/3900	39 (37, 42)	70 (67, 72)	54 (51, 57)	40 (36, 45)	39 (36, 43)	34 (31, 37)
Randomization arm							
Implementing	916/2417	39 (36, 43)	67 (63, 71)	53 (49, 58)	40 (34, 46)	38 (34, 43)	31 (28, 35)
Comparison	889/2515	38 (35, 41)	72 (69, 76)	55 (52, 58)	39 (34, 44)	38 (35, 42)	38 (34, 42)
Malaria prevalence (tertile)							
Low	511/1664	34 (30, 38)	76 (70, 82)	54 (49, 59)	31 (25, 38)	27 (23, 31)	39 (34, 44)
Medium	604/1615	39 (35, 44)	70 (66, 74)	55 (51, 59)	40 (33, 47)	36 (31, 42)	32 (28, 38)
High	690/1653	42 (39, 46)*	64 (60, 69)*	53 (48, 58)	48 (41, 56)*	51 (47, 55)	33 (30, 37)
Wealth index (tertile)							
Low	609/1677	38 (35, 41)	69 (66, 73)	52 (48, 57)	40 (34, 46)	38 (34, 43)	31 (26, 36)
Medium	644/1696	40 (37, 43)	69 (65, 74)	53 (49, 58)	43 (36, 51)	42 (37, 48)	36 (32, 40)
High	552/1559	37 (34, 40)	71 (65, 76)	56 (52, 62)	35 (30, 42)	33 (29, 39)	36 (31, 43)
Indoor residual spraying (IRS) area							
No IRS	1562/4054	41 (38, 43)*	68 (66, 71)*	54 (52, 57)	39 (36, 44)	39 (36, 42)	34 (31, 36)
IRS area	243/878	28 (25, 32)*	78 (70, 85)*	50 (42, 59)	39 (28, 53)	31 (26, 38)	40 (33, 48)

*p < 0.05 (based on prevalence ratios)

### Malnutrition

Malnutrition was relatively uncommon in the MVPE areas. Based on MUAC measurements, 5% (95%CI 4–6) of children were classified as at risk, 1% with moderate acute and < 1% with severe acute malnutrition (Supplemental Table S3). When combined, 6% (95%CI 5–7) were either at risk or acutely malnourished, with notable geographic heterogeneity (Table 5, Fig. 2E, Supplemental Table S4). The prevalence of malnutrition declined with increasing age and household wealth. Slightly higher rates were observed among girls compared to boys and in areas with moderate to high malaria prevalence. No differences were observed between urban and rural residence or by implementing and comparison areas.

**Table 5:** Mid-upper-arm circumference (MUAC) and prevalence of being at risk of malnutrition or acutely malnourished (MUAC ≤ 13.5cm) by population characteristics

Background Characteristics	Mean MUAC (SE), cm	Risk or presence of malnutrition (MUAC < 13.5cm)	
		% (95% CI) weighted	PR (95%CI)
Overall	15.66 (0.05)	6 (5, 7)	–
Age group			
5–11 months	15.00 (0.06)	13 (10, 17)	Reference
12–23 months	15.26 (0.06)	9 (7, 11)	0.67 (0.49, 0.91)*
24–35 months	15.70 (0.09)	6 (4, 8)	0.42 (0.27, 0.65)*
36–48 months	16.26 (0.06)	2 (1, 3)	0.12 (0.07, 0.21)*
Sex			
Female	15.58 (0.05)	7 (6, 9)	1.28 (1.01, 1.63)*
Male	15.74 (0.06)	6 (4, 7)	Reference
Residence			
Urban	15.68 (0.08)	6 (5, 9)	Reference
Rural	15.66 (0.06)	6 (5, 8)	0.98 (0.68, 1.41)
Randomization arm			
Implementing	15.70 (0.07)	6 (5, 7)	0.89 (0.63, 1.24)
Comparison	15.62 (0.07)	7 (5, 9)	
Malaria prevalence			
Low	15.73 (0.05)	5 (3, 7)	Reference
Medium	15.70 (0.09)	7 (5, 9)	1.42 (0.91, 2.23)
High	15.53 (0.09)	7 (6, 9)	1.52 (1.01, 2.28)*
Wealth Index			
Low	15.49 (0.07)	9 (7, 12)	Reference
Medium	15.63 (0.06)	6 (4, 7)	0.61 (0.44, 0.83)*
High	15.87 (0.06)	4 (3, 6)	0.46 (0.30, 0.72)*

*p < 0.05 (based on prevalence ratios)

### HBR, vaccination, vitamin A and deworming coverage

**Fig. 3:**
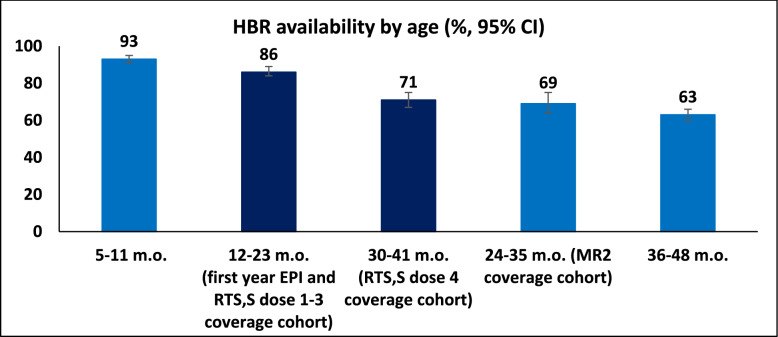
Home-based record availability by age, including relevant Expanded Programme on Immunization (EPI) age group (RTS,S-specific, first year of life, and measles dose 2)

HBR availability declined with increasing age (Fig. 3). Among children aged 12–23 months—the age cohort relevant for coverage of first-year EPI vaccines and RTS,S doses 1–3—HBRs were available for 86% (95% CI: 84–89), with lower retention in girls compared to boys (84% vs. 89%, p = 0.025). HBR availability declined to 71% (95% CI: 67–75) among those aged 30–41 months (RTS,S dose-4 cohort) and 69% (95% CI 63–74) among 24–35-month-olds (second dose of measles and rubella vaccine, MR2, cohort). Considerable geographic variation was observed, with sub-county-level HBR availability ranging from 69 to 100% (Fig. 2F; Supplemental Table S5). No significant differences in HBR availability were observed by urban versus rural residence, malaria prevalence tertile, randomization arm, or household wealth (Supplemental Table S6). However, slightly lower HBR availability was noted in areas implementing IRS.

**Fig. 4:**
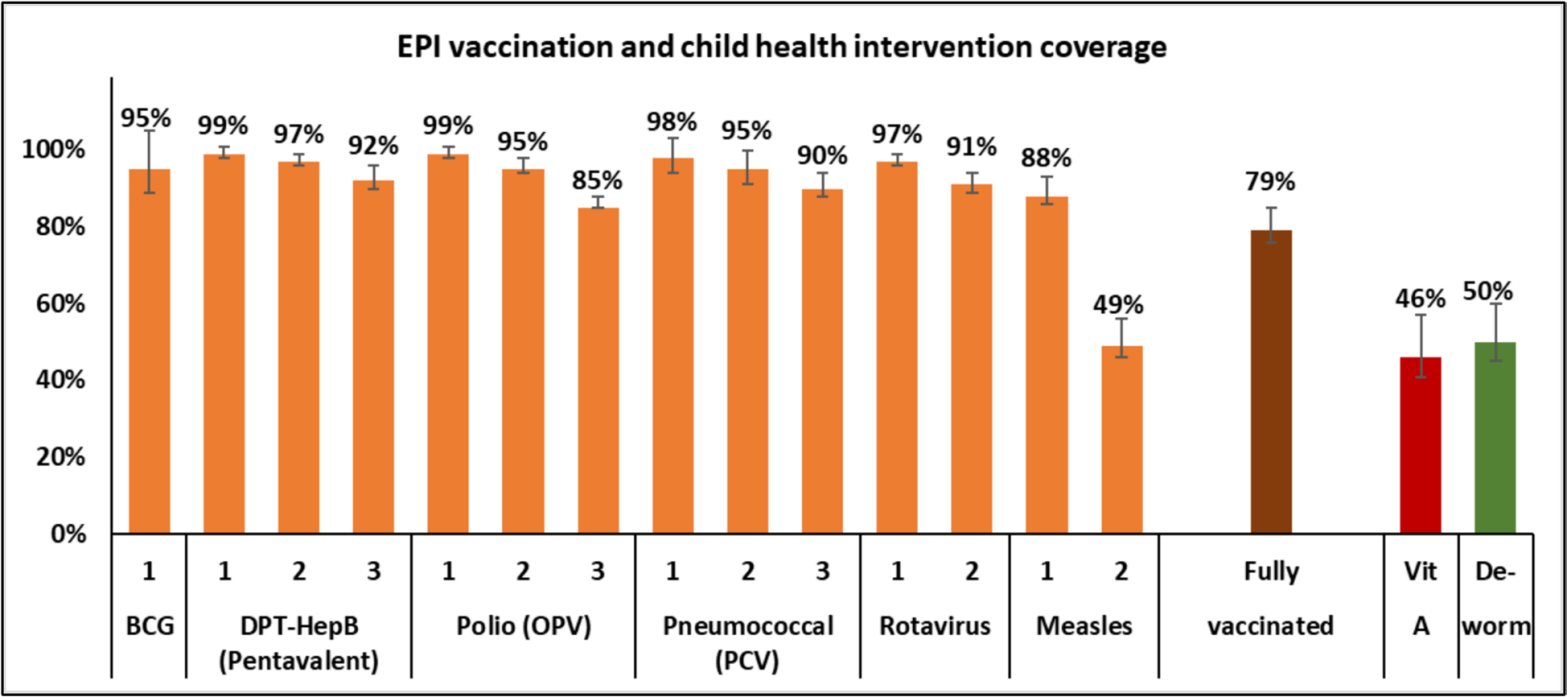
Expanded Programme on Immunization (EPI) Vaccination and child health interventions coverage by home-based record (HBR) or recall, if HBR is not available vaccination coverage is in children 12–23 months old (except for measles dose 2, 24–35 months); fully vaccinated is based on basic coverage (BCG, 3 doses of DTP-HepB, 3 doses of polio, and measles dose 1) in first year of life. Vitamin A coverage is based on HBR or recall, deworming by recall alone. Error bars represent 95% confidence intervals

Vaccination coverage by HBR or recall, when HBR was not available, was high (> 85%) for all vaccines, except for the second dose of measles vaccine (49%), which is administered in the second year of life (Fig. 4, Tables 6, 7). Coverage measurement restricted to children with available HBR was comparable (Supplemental Table S7), and additional coverage indicators based on alternative denominator definitions are presented in Supplemental Table S8. Substantial geographic variation in vaccination coverage was observed across MVPE Counties (Supplemental Table S9). No significant differences in coverage were detected by child sex, residence (except slightly higher pentavalent dose 3 coverage in urban compared to rural areas), randomization arm, malaria prevalence tertile, wealth (except for higher coverage in both measles doses in the highest compared to lowest tertiles), and IRS (except for considerably lower measles dose 2 coverage in IRS than non-IRS areas). Analysis of age at vaccination for pentavalent vaccine and measles dose-1– used as benchmark for the first year of life vaccines, including RTS,S doses 1–3—indicated that vaccinations were administered largely on schedule (Supplemental Figure S3).

**Table 6:** Vaccination coverage (home-based record [HBR] or recall, when HBR is not available), by population characteristics

Vaccine	Dose	Background characteristics							
		Overall		Sex		Residence		Arm	
				*Female*	*Male*	*Urban*	*Rural*	*Implementing*	*Comparison*
		n/N unweighted	% (95% CI) weighted	% (95% CI) weighted	% (95% CI) weighted	% (95% CI) weighted	% (95% CI) weighted	% (95% CI) weighted	% (95% CI) weighted
BCG	1	1105/1141	95 (89, 99)	97 (96, 99)	94 (89, 99)	91 (81, 100)	96 (95, 98)	95 (93, 98)	95 (91, 100)
DPT-HepB (Pentavalent)	1	1288/1304	99 (98, 1.00)	99 (99, 100)	99 (98, 100)	100 (100, 100)	99 (98, 99)	99 (98, 100)	99 (98, 100)
	2	1267/1304	97 (96, 98)	98 (97, 99)	96 (95, 98)	99 (98, 100)	97 (96, 98)	98 (96, 99)	97 (96, 98)
	3	1197/1304	92 (90, 94)	92 (89, 95)	92 (89, 94)	96 (93, 99)*	90 (88, 93)*	92 (90, 94)	91 (88, 95)
Polio (OPV)	1	1275/1289	99 (98, 1.00)	99 (99, 100)	98 (97, 100)	98 (96, 100)	99 (98, 100)	99 (98, 100)	98 (97, 100)
	2	1230/1289	95 (94, 97)	96 (95, 98)	94 (92, 97)	96 (94, 99)	95 (94, 96)	96 (95, 98)	94 (92, 96)
	3	1100/1289	85 (83, 88)	85 (81, 88)	86 (82, 89)	90 (87, 94)	84 (81, 87)	85 (81, 90)	85 (82, 88)
Pneumococcal (PCV)	1	1250/1282	98 (97, 99)	98 (97, 99)	97 (96, 98)	99 (97, 100)	98 (97, 98)	97 (96, 99)	98 (97, 99)
	2	1221/1282	95 (94, 96)	95 (93, 97)	94 (93, 96)	96 (94, 99)	95 (93, 96)	94 (92, 96)	96 (94, 97)
	3	1155/1282	90 (88, 92)	91 (88, 94)	90 (87, 92)	92 (88, 97)	90 (88, 92)	89 (86, 92)	92 (89, 94)
Rotavirus	1	1242/1283	97 (96, 98)	98 (96, 99)	96 (94, 98)	98 (95, 100)	97 (95, 98)	96 (94, 98)	98 (96, 99)
	2	1170/1283	91 (89, 92)	92 (89, 94)	90 (87, 92)	91 (87, 96)	90 (88, 93)	89 (86, 92)	92 (89, 94)
Measles	1	1162/1309	88 (86, 91)	90 (87, 92)	87 (83, 90)	92 (88, 97)	87 (84, 90)	85 (82, 89)	91 (88, 94)
	2	627/1243	49 (46, 53)	52 (47, 58)	47 (43, 52)	53 (46, 62)	48 (45, 53)	48 (44, 53)	51 (45, 57)
Fully vaccinated	(basic)	914/1141	79 (76, 82)	82 (78, 85)	76 (71, 82)	80 (73, 88)	78 (75, 82)	76 (72, 81)	81 (77, 86)

Vaccine coverage is measured in children 12–23 months old for all vaccinations in first year of life; measles dose 2 coverage is measured in children 24–35 months of age. * p < 0.05 based on coverage ratios

**Table 7:** Vaccination coverage (HBR or recall, when HBR is not available), by population characteristics

Vaccine	Dose	Background characteristics							
		Malaria prevalence (tertile)			Wealth (tertile)			Indoor residual spraying (IRS)	
		*Low*	*Middle*	*High*	*Low*	*Middle*	*High*	*No IRS*	*IRS*
		% (95% CI) weighted	% (95% CI) weighted	% (95% CI) weighted	% (95% CI) weighted	% (95% CI) weighted	% (95% CI) weighted	% (95% CI) weighted	% (95% CI) weighted
BCG	1	94 (87, 100)	97 (95, 99)	95 (91, 98)	97 (96, 99)	95 (91, 99)	94 (90, 97)	95 (92, 98)	98 (95, 100)
DPT-HepB (Pentavalent)	1	99 (98, 100)	99 (98, 100)	99 (98, 100)	99 (98, 100)	99 (98, 100)	99 (98, 100)	99 (99, 100)	98 (95, 100)
	2	97 (96, 99)	97 (95, 98)	98 (97, 99)	96 (94, 99)	97 (96, 99)	98 (97, 99)	98 (97, 99)	93 (89, 97)
	3	90 (86, 96)	92 (89, 94)	93 (91, 95)	91 (88, 94)	92 (89, 95)	92 (88, 97)	93 (91, 95)	84 (79, 90)
Polio (OPV)	1	98 (96, 100)	99 (98, 100)	100 (99, 100)	99 (99, 100)	98 (96, 100)	99 (98, 100)	99 (98, 100)	98 (95, 100)
	2	93 (91, 96)	95 (93, 97)	98 (96, 99)	96 (94, 98)	93 (90, 96)	97 (96, 99)	96 (95, 98)	90 (85, 95)
	3	85 (82, 88)	84 (79, 90)	87 (82, 91)	86 (81, 92)	83 (78, 87)	87 (83, 91)	87 (84, 89)	77 (71, 84)
Pneumococcal (PCV)	1	98 (97, 100)	97 (96, 99)	97 (96, 99)	97 (96, 99)	97 (96, 99)	99 (98, 100)	98 (97, 99)	96 (94, 99)
	2	96 (93, 99)	94 (92, 97)	95 (93, 97)	95 (92, 97)	94 (91, 97)	94 (91, 97)	95 (94, 97)	93 (88, 97)
	3	90 (86, 95)	89 (86, 92)	92 (90, 94)	89 (86, 92)	89 (86, 92)	92 (89, 96)	91 (89, 93)	85 (80, 91)
Rotavirus	1	98 (96, 100)	96 (93, 99)	97 (95, 99)	97 (95, 99)	96 (94, 98)	97 (95, 99)	97 (96, 98)	96 (93, 99)
	2	91 (87, 94)	89 (86, 93)	92 (89, 95)	91 (88, 94)	90 (87, 93)	92 (89, 94)	91 (89, 93)	88 (82, 94)
Measles	1	89 (86, 93)	84 (80, 90)	91 (88, 95)	82 (77, 87)*	89 (86, 92)	93 (90, 96)*	89 (87, 92)	82 (76, 89)
	2	51 (45, 58)	45 (40, 52)	52 (47, 59)	42 (36, 49)*	50 (45, 56)	55 (49, 62)*	52 (48, 56)*	39 (32, 47)*
Fully vaccinated	(ba-sic)	81 (75, 87)	77 (71, 82)	79 (75, 84)	74 (68, 80)	81 (75, 86)	81 (78, 86)	79 (76, 83)	75 (68, 83)

Vaccine coverage is measured in children 12–23 months old for all vaccinations in first year of life; measles dose 2 coverage is measured in children 24–35 months of age. * p < 0.05 based on coverage ratios

Coverage of vitamin A supplementation within the 6 months preceding the survey was 46% (95%CI 41–52) and deworming coverage was 50% (95%CI 45–55). No differences in coverage of these two child health interventions were observed by sex, randomization arm, malaria prevalence, or IRS implementation (Supplemental Table S10). However, vitamin A supplementation coverage was significantly lower in rural compared to urban areas (42% vs. 64%, p < 0.05). Both health interventions had higher coverage among the children in the highest household wealth group compared to those in the lowest (vitamin A: 55% vs. 37%; deworming: 66% vs. 37%; p < 0.05).

### Agreement between caregiver recall of vaccinations and HBR

Among children with HBR (n = 3768), comparison of vaccination coverage estimates derived from HBR versus caregiver recall demonstrated good agreement across all vaccines (any dose) as assessed by three metrics: percent agreement, Gwet’s AC1, and the Brennan-Prediger coefficient (Supplemental Table S11). Agreement levels were consistently high with AC1 values ranging from 0.74 to 0.97 and Brennan-Prediger coefficients ranging from 0.67 to 0.96. However, when disaggregated by specific vaccine dose, both coverage estimates and agreement metrics showed greater variability, largely attributable to inconsistencies in caregiver recall.

### Awareness and acceptability of malaria vaccine

We interviewed 4,169 caregivers of 4,948 children regarding their perceptions of malaria, awareness of the malaria vaccine, and willingness to vaccinate their children. Most caregivers perceived malaria as a serious health concern (80.6%), reported it as common in their community (78.2%), and believed that it predominantly affected children (85.9%). Just over one-third (36.3%) had heard of the malaria vaccine ahead of vaccine introduction, with the most commonly cited sources of information being radio (37.0%), community interviewers (25.1%), health facilities (19.0%), and community events (14.4%). Among those aware of the vaccine, the majority believed it was beneficial (93.1%), safe (88.0%), and offered some level of protection (89.4%) (Supplemental Figure S4). Before ascertaining acceptability, we provided a brief overview of the malaria vaccine to all caregivers, regardless of prior awareness of the vaccine. When asked “would you accept the new malaria vaccine for your child”, nearly all respondents (99%) indicated they would accept the vaccine for their child under each of the following conditions: if it was part of routine immunization, free of charge, safe, and available. Likewise, 99% stated they would bring the child for all four required doses. Acceptability remained high (98.3%) even when caregivers were reminded that malaria infection could still occur post-vaccination. Only 3.3% expressed hesitancy citing concerns related to vaccine efficacy, potential side effects, or time constraints.

## Discussion

The MVPE baseline feasibility survey, conducted just prior to the pilot introduction of RTS,S malaria vaccine in western Kenya, provides a comprehensive assessment of the demographic, epidemiological, and immunization landscape across 46 sub-counties which participated in MVIP. This survey characterized malaria prevalence and control measures, as well as caregivers’ health-seeking behaviors and access to interventions, with a level of detail that surpasses other existing national surveys and health surveillance data. The population-representative data serve as a foundational reference for evaluating subsequent changes in malaria burden and child health outcomes following the introduction of the malaria vaccine and other future interventions. Findings highlight both opportunities and challenges for successful malaria vaccine implementation and broader child health programming.

The survey, conducted from July to October 2019, reported *P. falciparum* malaria prevalence by RDT among children aged 5–48 months averaging 22% with marked geographic heterogeneity (1–71%), underscoring the high and uneven burden of malaria in the lake-endemic region. These findings are consistent with the 23% prevalence, also by RDT, reported by the MIS conducted in the same region in November–December 2020 and other studies [18–21]. Consistent with the findings in these studies, prevalence was highest in older children, rural areas, and among the lowest wealth tertiles, and was lower in areas where IRS had been implemented, suggesting the effectiveness of IRS in reducing malaria transmission. These patterns reinforce the need for continued, targeted malaria control efforts and highlight the potential added value of the malaria vaccine in high-burden settings, including where other interventions are in place but malaria in children remains a public health concern. The difference at baseline in malaria prevalence between study arms (26% in RTS,S implementing vs. 19% in comparison areas) suggests an imbalance in randomization that should be accounted for in the analyses of the midline and endline surveys and considered when interpreting impact evaluations. It may also reflect underlying differences in ecological, health system, or intervention coverage (e.g., greater number of clusters implementing IRS in the comparison arm, differential distribution of ITN types, etc.) factors that may confound comparisons between arms. Both pilot-based malaria vaccine impact evaluations in Kenya to date – on mortality and severe malaria from the MVPE [11, 22] and on outpatient malaria using routine surveillance data [23]– adjusted the analyses by RTS,S-ineligible age groups to account for cluster-level differences in malaria prevalence, interventions, and contextual factors, thereby controlling for baseline differences [24]. Future evaluations would benefit from adopting similar strategies, incorporating baseline malaria prevalence directly in adjusted models, and conducting sensitivity analyses to assess the robustness of impact estimates to baseline imbalances.

Ownership and use of malaria prevention and treatment interventions was encouraging but showed room for improvement. Household ITN ownership (93%) and use among children (87%) were high by regional standards, though gaps persisted among lower-income households. These findings are comparable to the most recent KDHS 2022 survey, which reported regional household ITN ownership at 91% and usage among children < 5 years at 78%, with sub-county ownership and use ranging 76–94% and 72–84%, respectively, across the 8 lake-endemic counties [25]. In contrast, KMIS 2020 reported ITN ownership by 78% of households, with 72% of children < 5 years using nets [19]. The lower estimates in KMIS 2020 are likely attributable to the attrition and deterioration of ITNs in context of delay in ITN mass distribution campaign from 2020 to 2021 due to COVID-19. The observed geographic variation in ITN coverage and use in our survey, as well as in the KMIS 2020 and KDHS 2022 data, suggests potential gaps in distribution or utilization that warrant further attention.

Comparable to other surveys, [19, 25] care-seeking for febrile illness was reported by 70% of caregivers, yet timely care-seeking and diagnostic testing lagged behind. These gaps in febrile illness management, particularly in high-prevalence sub-counties, highlight the importance of community education to address the issues of delayed care-seeking and the need for integrated approaches to fever case management, including community-level diagnostic and treatment access, which will be crucial for optimizing malaria vaccine impact.

The nutritional status of children was relatively favorable, with only 1% classified as moderately or severely acutely malnourished and 5% at risk of malnutrition, similar to other surveys [25]. However, consistent with other studies, [26] malnutrition remained more common in younger children, poorer households, and high-malaria areas, suggesting continued vulnerability in overlapping domains of child health. These findings support the integration of nutrition services with malaria and immunization programming, particularly for children at highest risk.

Coverage of vitamin A supplementation and deworming (46% and 50%, respectively) was lower in our survey than the Kenya national estimates (63% and 65%, respectively; no regional data available), however trends for lower coverage of both interventions among rural and poorer households reported in KDHS 2022 [25], as well as geographic heterogeneity, were observed in our survey as well. These findings suggest that expanding and integrating the malaria vaccine platform into immunization systems could provide an opportunity to strengthen delivery of other non-vaccine child health services, particularly when these are delivered during routine contacts aligned with malaria vaccine visits. This includes interventions scheduled at similar ages, such as vitamin A supplementation at 6 month intervals (e.g., alongside RTS,S dose 1 at 6 months and dose 4 at 24 months) and deworming at 24 months.

Availability of HBR was suboptimal overall (76%) and declined with child age, potentially impacting vaccine documentation and defaulter tracing. This pattern is consistent with findings from Kenya 2022 DHS [25], where the national proportion of children with HBR decreased from ~ 75% among children aged 12–23 months to ~ 60% among those aged 24–35 months. As a result, measurement of vaccination coverage for doses administered later in the schedule increasingly relies on caregiver recall. This has important implications for multi-dose, time-sensitive vaccines such as RTS,S, particularly for dose 4 administered in the second year of life. Lower HBR availability in older children may lead to greater misclassification of coverage for later doses in both survey-based and routine monitoring systems. Although agreement between caregiver recall and HBR for vaccination history was high at the aggregate level, dose-specific recall tended to underestimate coverage, suggesting the need for strengthened documentation practices, especially as Kenya prepares for the expansion of multi-dose, time-sensitive vaccines like RTS,S.

Vaccination coverage for most vaccines delivered in the first year of life was high (> 85%, with 79% of children fully vaccinated), consistent with national data and confirming a relatively well-functioning routine immunization system. [25] In contrast, coverage of measles second dose (49%)—introduced in 2014 and administered at 18 months of age—was markedly lower, especially in IRS-implementing areas. These findings align with existing evidence on the challenges of sustaining immunization engagement beyond infancy [27, 28], and suggest that introducing a four-dose malaria vaccine extending into the second year of life may require targeted strategies to reinforce contact with health services over time [14]. Notably, no major differences in vaccine coverage were observed by child’s sex, residence, or malaria burden tertile—an encouraging signal of equity in the existing immunization platform.

Consistent with a concurrent in-depth qualitative evaluation of malaria vaccine introduction in Kenya, [29] caregivers’ acceptance of the malaria vaccine was high, even after receiving balanced information about the vaccine’s partial efficacy. High intent to complete the full four-dose series and low levels of hesitancy indicate a favorable baseline for RTS,S implementation. However, the relatively low baseline awareness of the vaccine (~ 36%) indicates insufficient local sensitization several months ahead of RTS,S introduction and highlights the need for continued communication and community engagement to sustain demand and ensure uptake, particularly in areas with limited health communication infrastructure.

This evaluation has several strengths, including large sample size, high response rates, population representativeness, and granular data that provide higher resolution at the lake-endemic region level than national surveys such as the DHS and MIS. However, some limitations warrant consideration. First, estimates of vaccination coverage may be subject to recall bias where HBRs were unavailable, although agreement between sources was generally high. Second, the survey provides point estimates for malaria prevalence and intervention coverage, but these outcomes may vary seasonally and over time. Further the timing and duration of the survey, between the end of the peak malaria season and the start of short rains, may have introduced heterogeneity into malaria prevalence. That said, we have examined the monthly malaria prevalence estimates, complicated by geographic patterns of data collection over time, and did not see significant differences or patterns over the data collection period. The small number of EAs within each implementation and comparison cluster (sub-county) increased the likelihood that estimates were not fully representative at the sub-county level, compared with a design including more EAs per cluster. However, the large total number of clusters included in the survey likely mitigated this concern for estimates at MVIP area level. Finally, the cross-sectional nature of the survey limits our ability to infer causal relationships between interventions and outcomes.

In conclusion, this baseline survey establishes a robust pre-intervention benchmark for the RTS,S malaria vaccine pilot in Kenya. The findings underscore persistent malaria burden, gaps in intervention access and coverage, well-functioning EPI system but with recognized challenges of achieving high coverage of second-year-of-life vaccinations, and strong caregiver support for malaria vaccination. These results will be critical for interpreting future changes in vaccine uptake and malaria outcomes, and for informing strategies to strengthen integrated child health service delivery in high-transmission settings.

## Supplementary Information

Supplementary material 1

## Data Availability

Anonymised data will be made available through Harvard Dataverse. Requests for access will be reviewed by a data access committee.
