# Supplementary material for: The Malaria Vaccine Programme Evaluation in Kenya: results of a baseline household survey prior to the introduction of the RTS,S/AS01 vaccine
Source: Malar J. 2026 Apr 21;25:198. doi: 10.1186/s12936-026-05911-2 (PMC13147653; doi:10.1186/s12936-026-05911-2)
Supplement: Supplementary file 1 — Supplementary material 1 [file 12936_2026_5911_MOESM1_ESM.docx]

# *The Malaria Vaccine Programme Evaluation in Kenya: results of a baseline household survey prior to the introduction of the RTS,S/AS01 vaccine*

## Supplementary materials

**Detailed Methods**

A detailed description of the demographic, epidemiologic, and health systems context for malaria vaccine introduction is provided in Annex 1. The details of the pilot design and cluster selection have been described elsewhere. [1, 2] The objective of the baseline feasibility survey was to characterize the MVIP areas and target population prior to vaccine introduction, assess balance across arms, and serve as a baseline for measuring the effect of malaria vaccine introduction on coverage and use of child health services and malaria prevention interventions. Specifically, the survey assessed coverage of recommended EPI vaccines, coverage and utilization of key malaria control and child health interventions (including deworming and vitamin A supplementation), nutritional status, and health-seeking behavior. The survey was conducted across all 46 MVIP sub-counties in western Kenya, comprising 23 sub-counties implementing the RTS,S malaria vaccine and 23 comparison sub-counties (Figure 1), each contributing an annual cohort of approximately 4,000 children for the evaluation. These clusters span eight counties—Bungoma, Busia, Homa Bay, Kakamega, Kisumu, Migori, Siaya, and Vihiga—that together constitute the lake-endemic zone, home to an estimated 9.8 million people in 2019 and characterized by high malaria transmission.

#### Sample size and sampling

The baseline survey employed a two-stage cluster sampling design. In each of the 46 MVIP sub-counties four enumeration areas (EAs) were selected by the Kenya National Bureau of Statistics (KNBS) using probability proportional to estimated population size. Within each selected EA, 25 households were randomly selected without replacement, targeting 100 households per sub-county. Each household had a known, non-zero probability of selection.

A sample size of 100 households per sub-county allowed estimation of cluster-specific coverage indicators with a precision of 10–15%, assuming a conservative estimate of 50% coverage and a high response rate (>95%) per cluster. In the 23 implementing sub-counties, a total sample of 2,300 households (25 per EA across 92 EAs), assuming a design effect of 1.5, provided an overall precision of ±2–3% around a true coverage estimate of 50%.

Following EA selection and prior to the survey, mapping teams trained in enumeration procedures delineated EA boundaries using household census maps with guidance from KNBS, and enumerated all households within each EA. During mapping, teams identified households with potentially eligible children based on age (<4y). These households formed the sampling frame within each EA. All consenting mothers or caregivers of children aged 5 to 48 months residing in selected households were eligible to participate. If there were multiple eligible children in a household, all children were included. Sampling weights were calculated based on the population size of each EA, non-response adjustment, and the probability of household selection.

#### Community sensitization

Extensive community sensitization activities were conducted prior to the start of the survey. These efforts were aligned with broader MVIP activities and carried out in collaboration with the MoH, EPI, and MVIP programme stakeholders. Initial meetings were held with regional and County commissioners, the County and sub-County Health Management Teams and government administrative officials to introduce the survey, outline its objectives, and provide an overview of planned activities and timelines. Following EA selection, additional meetings were held with local leadership in each selected EA. These included chiefs, assistant chiefs, village elders, community health volunteers (CHVs), and other community leaders.

#### Data collection

Data collection was conducted over a 13-week period from July to October 2019. Twelve field teams comprising of a team lead and community interviewers were each assigned to approximately four sub-counties (16 EAs). Teams were allocated based on local language competencies to ensure effective communication across the pilot implementation area in western Kenya.

The baseline survey methodology was adapted from standard household survey protocols routinely used in Kenya, including the Demographic and Health Surveys (DHS), Malaria Indicator Surveys (MIS), and Multiple Indicator Cluster Surveys (MICS), in consultation with KNBS. A two-week training was conducted for team supervisors, interviewers, monitors, and quality assurance staff, following the DHS training curriculum. The training concluded with a 3-day field pilot of the questionnaires during which translations were reviewed and refined based on field experience.

Prior to data collection activities, local administration and CHVs in selected villages and EAs were reminded about the upcoming survey. Field teams were provided with details of the selected EAs and sampled households. A village guide accompanied field teams to ensure accurate identification of households. For each selected household, at least two callbacks were made if eligible respondents were initially unavailable. Each team spent approximately three days per EA, with additional time allocated for tracing households not available during the initial visits. Three regional field supervisors and three data monitors were based in hubs covering the northern, central, and southern regions of the study area. These staff conducted daily visits to EAs to monitor field activities and ensure adherence to protocols. Field coordinators, study coordinators, and study investigators also provided regular supervision through field visits to support and oversee data collection.

#### Informed consent

Written informed consent was obtained from the head of each household for participation in the study and from all mothers or primary caregivers of eligible children. Consent included permission for the child’s participation in the survey, as well as for malnutrition assessment through MUAC measurement, malaria testing, treatment, and referral for severe malaria or malnutrition care when needed. Caregivers had an option to respond to the questionnaire but opt out of child’s malaria testing and malnutrition assessment.

All consenting mothers or primary caregivers of children within the eligible age range were interviewed. A separate interview was conducted for each eligible child; therefore, some caregivers were interviewed more than once. In accordance with Kenya's national guidelines for HIV counseling and testing [3], mothers under the age of 18 were considered “mature minors” and were able to provide consent for themselves and their children. Consent forms were translated into the Dholuo, Maragoli, Bukusu, and Kiswahili languages and back translated into English to ensure accuracy. Participant confidentiality was strictly maintained throughout all stages of the research process.

**Questionnaires**

To facilitate comparability and interpretation of results with previous national surveys we adapted quantitative variables from standardized instruments used in DHS, MIS, and MICS. Additional variables were incorporated to align with the specific MVPE objectives. Questions assessing vaccine acceptability were drawn from Knowledge, Attitudes, and Practices (KAP) surveys previously implemented for cholera, measles, influenza, and other vaccines, as well as from the MVIP Health Utilization Study.[4] The questionnaires were grouped in four different domains: (1) household information: household location, demographic composition, socio-economic status, and access to malaria prevention; (2) mother/caregiver data: demographics and birth history; (3) child health data: child demographics, immunization history, use of malaria control and other interventions, health seeking behavior for febrile illness, malaria testing and treatment, and nutritional status; (4) vaccine acceptability: perceptions on malaria, acceptability of RTS,S vaccine. Questionnaires (Supplementary materials, Annex 2) were developed in English and translated into the local languages of Kiswahili, Dholuo, Maragoli, and Bukusu and back translated to English for accuracy.

**Immunization history**

Immunization history was assessed by requesting to see the home-based immunization record (HBR; Mother and Child Health Booklet in Kenya) to capture vaccinations for each eligible child. If available, dates of all recorded vaccinations were transcribed, and a photo of the HBR was taken. Dose numbering was based on number of doses received, rather than dose timing. When a vaccination was not recorded, the caregiver was asked to recall whether the child received the vaccine. If the HBR was not available, caregivers were asked to recall all vaccinations and number of doses received. An MoH-developed job aid (Supplementary materials, Figure S1) showing different vaccines by age, as well as standardized verbal descriptions of the vaccine administration mode, site, and timing were used to facilitate the caregiver recall. To assess the reliability of caregiver recall, mothers were asked to verbally recall their child’s vaccination history even if the HBR was available.

**Nutritional assessment**

Nutritional status was assessed using a color-coded MUAC tape to measure the circumference of the left upper arm at its midpoint. Malnutrition status was defined as at risk if MUAC was 12.5cm to <13.5cm, moderate acute malnutrition MUAC 11.5cm to <12.5cm, and severe acute malnutrition MUAC <11.5cm. Due to low numbers of children with acute malnutrition, malnutrition levels were combined into a “risk or presence of malnutrition” variable for MUAC <13.5cm for some analyses. Children identified as malnourished were referred to the nearest health facility for further assessment and care in accordance with the Kenya National Guidelines for Integrated Management of Acute Malnutrition. [5]

**Malaria testing and treatment**

Capillary blood samples were obtained by finger prick to test for *Plasmodium* spp. infection using SD Bioline Malaria Ag P.f/Pan RDT (product code 05FK60; Standard Diagnostics, Inc., Republic of Korea). Children who tested positive by either P.f. or Pan band were treated on site in accordance with Kenya’s National Malaria Treatment Guidelines.[6] Children exhibiting signs of severe malaria or serious illness were referred to the nearest health facility and the local CHV was assigned to follow-up.

#### Data management and analysis

Survey data were collected electronically using tablet computers equipped with Open Data Kit (ODK) software. Questionnaires were geo-coded and time-stamped to support quality assurance, including tracking of interview duration and verification of field activity locations. Built-in validation rules within ODK flagged missing values, outliers, inconsistencies, and other discrepancies in real time. Upon completion of each questionnaire, team leads conducted real-time checks for missing responses and inconsistencies. Encrypted data were then transferred to a secure cloud server with local backups maintained. A second level of data quality control was conducted by data monitors based at regional hubs who reviewed submissions for discrepancies or missing data and coordinated timely corrections before submission to the central database.

All analyses were weighted and accounted for clustering. Descriptive statistics were generated using the survey analysis procedures in SAS v9.4 and Stata v18 estimating immunization coverage, health-seeking behavior, intervention uptake, and other key indicators. Analyses were stratified by sex, residence (urban/rural), RTS,S implementing and comparison area, socio-economic-status (SES), and malaria prevalence tertiles. SES tertiles were derived using principal component analysis of household assets. Following standard EPI coverage assessment methodology, coverage of vaccines scheduled during the first-year-of-life was estimated among children aged 12–23 months, while coverage for vaccines scheduled during the second year of life was estimated among children aged 24–35 months. For the RTS,S vaccine specifically, we applied additional age strata relevant to RTS,S vaccine coverage assessment to facilitate comparability across various indicators with midline and endline surveys: age stratum relevant to RTS,S doses 1–3 was 12–23 months, consistent with other first-year-of-life vaccines; age stratum relevant to RTS,S dose 4 was 30–41 months, reflecting its administration at 24 months with a 6-month interval before evaluation. Additionally, vaccine coverage was calculated based on availability of vaccination records in two ways: 1) by HBR or caregiver recall, if HBR was not available (primary); and 2) by HBR alone (secondary). The analysis evaluating the agreement between the caregiver recall and HBR was carried out on a subset of children for whom both HBR and recall were provided. Agreement between recall and HBR was assessed using percent agreement, Brennan-Prediger coefficients, and Gwet’s AC1 coefficients. An agreement coefficient >0.70 was considered indicative of acceptable concordance.

#### Ethical considerations

The Kenya country-specific MVPE protocol and associated study materials received ethical approval from the following institutions: the Kenya Medical Research Institute (KEMRI) Scientific and Ethical Review Unit (SERU) (Protocol #: 3771), the U.S. Centers for Disease Control and Prevention (CDC) Institutional Review Board (IRB) (Protocol #: 7184), the Oxford Tropical Research Ethics Committee (OxTREC) (Protocol #: 55-18), and the World Health Organization (WHO) Ethical Review Committee (ID: Kenya RTS,S MVIP).

**References**

1. Asante, K.P., et al., *Feasibility, safety, and impact of the RTS,S/AS01(E) malaria vaccine when implemented through national immunisation programmes: evaluation of cluster-randomised introduction of the vaccine in Ghana, Kenya, and Malawi.* Lancet, 2024. **403**(10437): p. 1660-1670.

2. Jalang'o, R., et al., *Subnational introduction of the RTS,S/AS01(E) malaria vaccine into routine immunization: experience and lessons from the three pilot countries.* Malar J, 2025. **24**(1): p. 244.

3. Ministry of Health (Kenya), N.A.a.S.C.P.N., *The Kenya HIV Testing Services Guidelines, 3rd Edition.* 2015.

4. Hill, J., et al., *Integration of the RTS,S/AS01 malaria vaccine into the Essential Programme on Immunisation in western Kenya: a qualitative longitudinal study from the health system perspective.* Lancet Glob Health, 2024. **12**(4): p. e672-e684.

5. Ministry of Health, K., *National Guidelines for the Integrated Management of Acute Malnutrition.* [*http://nak.or.ke/wpcontent/uploads/2017/12/Kenya-MoH-IMAM-Guideline-June-2009.pdf*](http://nak.or.ke/wpcontent/uploads/2017/12/Kenya-MoH-IMAM-Guideline-June-2009.pdf). 2009.

6. Programme, M.o.H.D.o.N.M., *Guidelines for the diagnosis, treatment, and prevention of malaria in Kenya 6th Edition*. 2020.

**Figure S1. MoH Immunization Schedule Poster used as job aid to facilitate recall**

**Figure S2. Malaria prevalence calculated per month of data collection (July-October 2019), including the total data collection days per month, proportion of the overall study sample that was tested each month, number of clusters and counties where the data collection was ongoing in each month.**

**
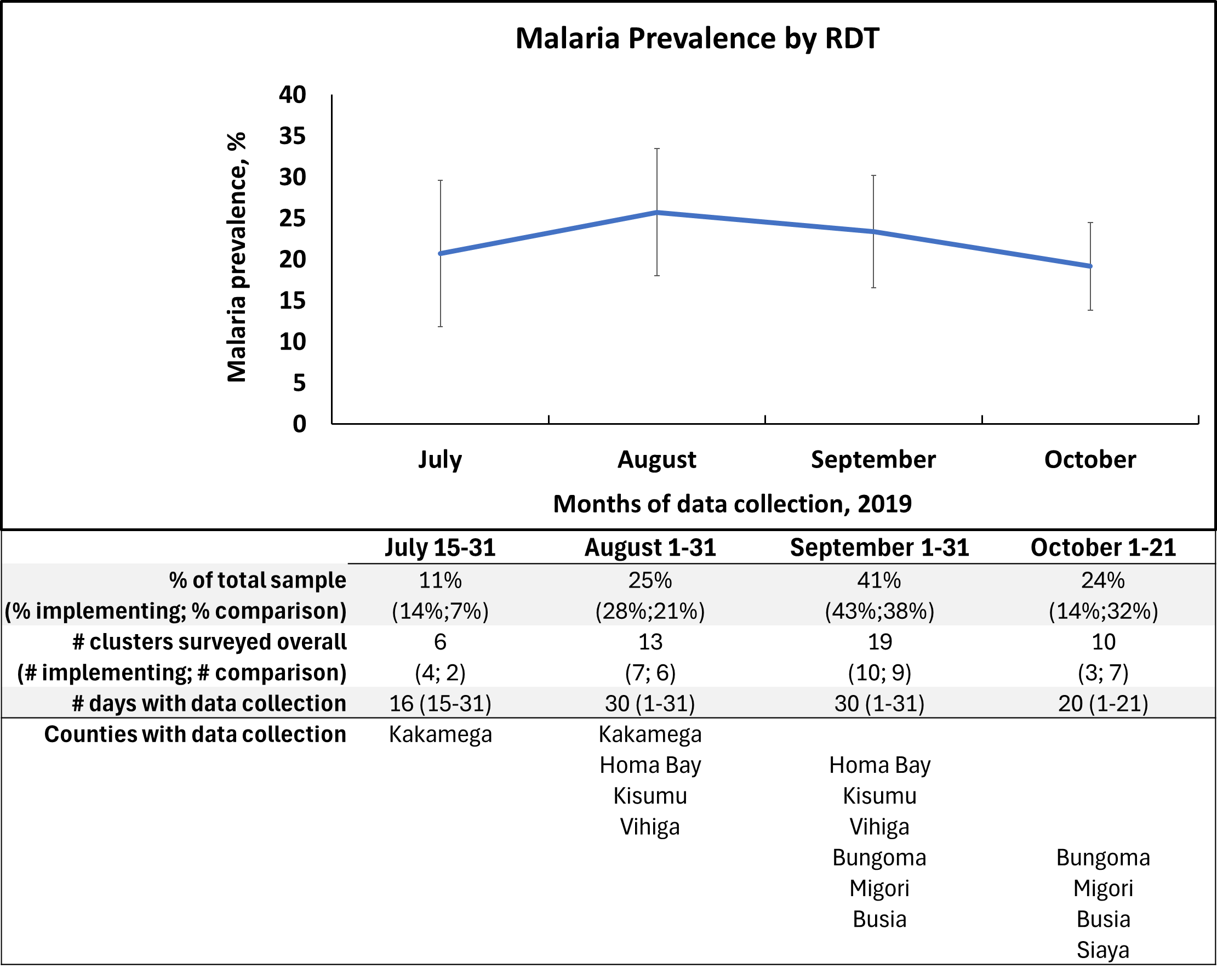
**

**Figure S3. Timing of Pentavalent doses 1-3 and Measles dose 1 based on age at the time of vaccination analysis (children with HBR only, 12-23 months)**

**Figure S4. Perceptions of malaria vaccine by the caregivers/mothers of children 5-48 m.o.**

**
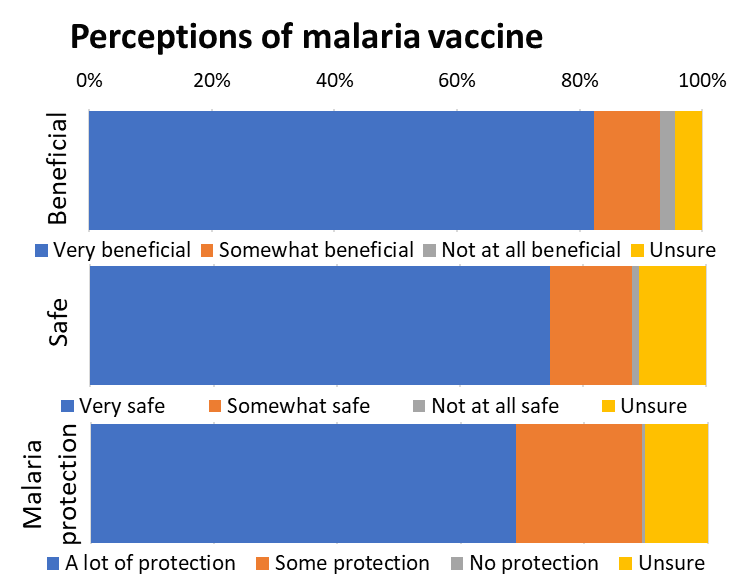
**

**Table S1. Household enrollment description**

| **Sample description** |  | | |
| --- | --- | --- | --- |
|  | **Implementing**  **areas** | **Comparison areas** | **Total** |
| **Number of households enumerated** | 13,515 (50%) | 13,432 (50%) | **26,947** |
| **Households selected for enrollment** | 2,885 (50%) | 2,938 (50%) | **5,823** |
| **Households refusing participation (%, out of eligible)** | 18 (0.7%) | 20 (0.8%) | **38 (0.7%)** |
| **Households interviewed (%, out of selected)** | 2,007 (70%) | 2,058 (70%) | **4,065 (70%)** |
| **Mothers/caregivers of children aged 5-48 months interviewed** | 2,067 (50%) | 2,102 (50%) | **4,169** |
| **Number of children enrolled, aged 5–48 months** | 2,430 (49%) | 2,518 (51%) | **4,948** |
| **Average number of children per household** | 1.21 | 1.22 | **1.22** |
| **Children consented to biomarkers (malaria testing and MUAC), % out of enrolled** | 2,400 (99%) | 2,488 (99%) | **4,888 (99%)** |

**Table S2. Malaria prevalence and prevention coverage at the county and sub-county level**

| **Name** | **MVIP Allocation** | **Malaria prevalence, % (95%CI)** | **Net ownership (≥1 per HH), % (95%CI)** | **Net use last night (children), %(95%CI)** | **IRS** |
| --- | --- | --- | --- | --- | --- |
| **Bungoma** |  | **27 (17 - 37)** | **91 (87 - 95)** | **85 (79 - 90)** |  |
| Bumula | Implementing | 45 (31-60) | 88 (82-93) | 82 (74-91) | 0 |
| Kanduyi | Comparison | 14 (8-20) | 95 (95-96) | 89 (88-91) | 0 |
| Sirisia | Comparison | 23 (13-33) | 89 (82-96) | 83 (70-95) | 0 |
| **Busia** |  | **45 (37 - 53)** | **97 (96 - 98)** | **91 (89 - 94)** |  |
| Budalangi | Comparison | 53 (38-68) | 97 (94-100) | 92 (85-100) | 0 |
| Butula | Implementing | 63 (55-70) | 96 (95-97) | 90 (86-94) | 0 |
| Funyula | Implementing | 59 (40-79) | 94 (91-97) | 89 (83-94) | 0 |
| Matayos | Comparison | 24 (9-39) | 98 (96-100) | 96 (92-99) | 0 |
| Nambale | Implementing | 46 (31-61) | 97 (94-100) | 91 (88-95) | 0 |
| Teso North | Comparison | 32 (22-41) | 98 (96-100) | 91 (84-99) | 0 |
| Teso South | Comparison | 53 (32-73) | 97 (94-100) | 89 (82-96) | 0 |
| **Homa Bay** |  | **5 (0 - 11)** | **90 (87 - 94)** | **83 (77 - 89)** |  |
| Homa Bay | Implementing | 2 (0-5) | 94 (86-100) | 87 (77-96) | 61 (55-66) |
| Kabondo Kasipul | Implementing | 2 (0-4) | 98 (94-100) | 91 (84-98) | 55 (40-70) |
| Karachuonyo | Comparison | 3 (0-5) | 93 (87-98) | 91 (87-96) | 53 (34-72) |
| Kasipul | Comparison | 1 (0-2) | 95 (93-97) | 89 (82-95) | 45 (35-56) |
| Ndhiwa | Implementing | 13 (0-33) | 83 (79-88) | 69 (61-77) | 56 (50-63) |
| Rangwe | Comparison | 1 (0-3) | 91 (84-97) | 88 (85-90) | 58 (47-69) |
| Mbita (Suba North) | Comparison | 3 (1-4) | 94 (92-96) | 91 (90-93) | 46 (39-53) |
| Suba (Suba South) | Comparison | 3 (0-8) | 90 (82-97) | 86 (77-95) | 70 (51-89) |
| **Kakamega** |  | **31 (23 - 40)** | **95 (93 - 97)** | **89 (86 - 92)** |  |
| Butere | Implementing | 40 (25-55) | 93 (87-99) | 84 (77-91) | 0 |
| Ikolomani | Implementing | 17 (10-24) | 94 (91-97) | 90 (86-94) | 0 |
| Khwisero | Implementing | 45 (30-60) | 91 (83-99) | 86 (78-95) | 0 |
| Lurambi | Comparison | 7 (0-14) | 97 (93-100) | 96 (91-100) | 0 |
| Malava | Implementing | 20 (14-26) | 100 (100-100) | 95 (92-98) | 0 |
| Matungu | Comparison | 71 (64-77) | 92 (90-95) | 87 (78-96) | 0 |
| Mumias East | Comparison | 47 (41-53) | 94 (89-99) | 82 (77-87) | 0 |
| Mumias West | Implementing | 49 (31-67) | 88 (80-95) | 79 (63-95) | 0 |
| Navakholo | Implementing | 49 (40-59) | 94 (88-100) | 88 (83-94) | 0 |
| Shinyalu | Comparison | 6 (1-11) | 94 (85-100) | 85 (81-89) | 0 |
| **Kisumu** |  | **12 (3 - 21)** | **95 (92 - 98)** | **93 (88 - 97)** |  |
| Kisumu Central | Implementing | 4 (0-8) | 94 (91-97) | 95 (92-97) | 0 |
| Kisumu East | Implementing | 6 (3-10) | 99 (96-100) | 98 (96-100) | 0 |
| Muhoroni | Implementing | 30 (8-52) | 92 (83-100) | 86 (73-99) | 0 |
| Nyakach | Comparison | 6 (2-10) | 96 (93-99) | 92 (89-96) | 0 |
| **Migori** |  | **8 (3 - 12**) | **93 (90 - 95)** | **86 (83 - 89)** |  |
| Awendo | Implementing | 3 (1-6) | 91 (87-95) | 82 (69-95) | 54 (46-62) |
| Nyatike | Implementing | 11 (3-19) | 94 (89-99) | 87 (80-93) | 53 (41-65) |
| Rongo | Comparison | 2 (0-3) | 93 (90-96) | 88 (81-95) | 56 (41-71) |
| Suna East | Comparison | 19 (8-29) | 96 (96-96) | 90 (87-92) | 60 (54-65) |
| Suna West | Comparison | 6 (0-19) | 93 (89-97) | 91 (87-94) | 39 (28-50) |
| Uriri | Comparison | 3 (0-7) | 87 (84-91) | 78 (73-82) | 69 (61-77) |
| **Siaya** |  | **40 (29 - 50)** | **92 (89 - 95)** | **87 (82 - 92)** |  |
| Rarieda | Implementing | 20 (12-28) | 96 (93-98) | 93 (90-95) | 0 |
| Ugenya | Implementing | 57 (46-68) | 90 (84-95) | 79 (75-83) | 0 |
| Ugunja | Comparison | 39 (34-44) | 92 (89-94) | 93 (91-95) | 0 |
| **Vihiga** |  | **12 (6 - 18)** | **89 (86 - 93)** | **84 (80 - 88)** |  |
| Emuhaya | Implementing | 22 (7-36) | 93 (87-98) | 86 (78-94) | 0 |
| Hamisi | Comparison | 9 (2-15) | 86 (76-95) | 79 (73-85) | 0 |
| Luanda | Implementing | 14 (3-26) | 87 (83-90) | 82 (74-91) | 0 |
| Sabatia | Comparison | 9 (0-27) | 93 (89-97) | 92 (86-97) | 0 |
| Vihiga | Implementing | 8 (4-12) | 89 (85-92) | 81 (77-85) | 0 |

**Table S3. MUAC and prevalence of malnutrition, by at risk, moderate acute, severe acute, and cumulative (risk or presence of malnutrition) categorization and population characteristics.**

| **Background Characteristics** | **Mean MUAC (SE), cm** | **At risk of malnutrition (12.5cm≤ MUAC <13.5cm)** | | **Moderate acute malnutrition  (11.5cm≤ MUAC <12.5cm)** | | **Severe acute malnutrition  (MUAC <11.5cm)** | |
| --- | --- | --- | --- | --- | --- | --- | --- |
|  |  | **% (95% CI) weighted** | **PR (95%CI)** | **% (95% CI) weighted** | **PR (95%CI)** | **% (95% CI) weighted** | **PR (95%CI)** |
| **Overall** | 15.66 (0.05) | 5 (4, 6) | - | 1 (1, 1) | - | 0 (0, 1) | - |
| **Age group** |  |  |  |  |  |  |  |
| 5-11 m.o. | 15.00 (0.06) | 11 (8, 14) | Reference | 2 (1, 3) | Reference | 1 (0, 1) | Reference |
| *12-23 m.o.* | 15.26 (0.06) | 7 (5, 9) | 0.65 (0.46, 0.92)* | 1 (1, 3) | 0.82 (0.39, 1.72) | 0 (0, 1) | 0.53 (0.13, 2.09) |
| 24-35 m.o. | 15.70 (0.09) | 4 (3, 6) | 0.37 (0.23, 0.59)* | 1 (0, 2) | 0.34 (0.11, 1.13) | 1 (0, 3) | 1.53 (0.34, 6.89) |
| 36-48 m.o. | 16.26 (0.06) | 1 (1, 3) | 0.13 (0.07, 0.25)* | 0 (0, 1) | 0.06 (0.01, 0.43) | 0 (0, 0) | 0.09 (0.01, 0.79) |
| **Sex** |  |  |  |  |  |  |  |
| Female | 15.58 (0.05) | 6 (5, 7) | 1.35 (1.04, 1.75)* | 1 (1, 1) | 1.22 (0.70, 2.10) | 0 (0, 1) | 0.65 (0.19, 2.20) |
| Male | 15.74 (0.06) | 4 (3, 6) | Reference | 1 (1, 1) | Reference | 0 (0, 1) | Reference |
| **Residence** |  |  |  |  |  |  |  |
| Urban | 15.68 (0.08) | 6 (4, 8) | Reference | 1 (1, 1) | 2.55 (1.03, 6.36) | 0 (0, 1) | Reference |
| Rural | 15.66 (0.06) | 5 (4, 6) | 0.86 (0.57, 1.30) | 0 (0, 1) | Reference | 0 (0, 1) | 1.18 (0.33, 4.30) |
| **Randomization arm** | |  |  |  |  |  |  |
| Implementing | 15.70 (0.07) | 5 (4, 6) | 0.93 (0.61, 1.40) | 1 (1, 1) | 0.83 (0.44, 1.60) | 0 (0, 0) | 0.51 (0.15, 1.71) |
| Comparison | 15.62 (0.07) | 5 (4, 7) | Reference | 1 (1, 2) | Reference | 0 (0, 1) | Reference |
| **Malaria prevalence** | |  |  |  |  |  |  |
| Low | 15.73 (0.05) | 4 (3, 6) | Reference | 1 (0, 1) | Reference | 0 (0, 0) | Reference |
| Medium | 15.70 (0.09) | 6 (4, 8) | 1.41 (0.83, 2.40) | 1 (1, 2) | 1.23 (0.50, 3.00) | 0 (0, 1) | 2.79 (0.54, 14.36) |
| High | 15.53 (0.09) | 6 (4, 8) | 1.49 (0.91, 2.42) | 1 (1, 2) | 1.49 (0.64, 3.50) | 0 (0, 1) | 2.51 (0.57, 11.12) |
| **Wealth Index** | |  |  |  |  |  |  |
| Low | 15.49 (0.07) | 7 (5, 9) | Reference | 2 (1, 2) | Reference | 0 (0, 1) | Reference |
| Medium | 15.63 (0.06) | 4 (3, 6) | 0.62 (0.44, 0.88)* | 1 (0, 1) | 0.46 (0.24, 0.87)* | 0 (0, 1) | 0.89 (0.16, 4.81) |
| High | 15.87 (0.06) | 4 (2, 6) | 0.50 (0.31, 0.83)* | 0 (0, 1) | 0.24 (0.10, 0.55)* | 0 (0, 1) | 0.65 (0.21, 2.05) |

**Table S4. Proportion of children at risk of malnutrition (MUAC ≤ 13.5cm)**

| **Name** | **MVIP Allocation** | **At risk of malnutrition, %(95%CI)** |
| --- | --- | --- |
| **Bungoma** |  |  |
| Bumula | Implementing | 7 (0-14) |
| Kanduyi | Comparison | 7 (0-13) |
| Sirisia | Comparison | 15 (5-25) |
| **Busia** |  |  |
| Budalangi | Comparison | 3 (0-6) |
| Butula | Implementing | 6 (0-11) |
| Funyula | Implementing | 9 (5-12) |
| Matayos | Comparison | 4 (1-7) |
| Nambale | Implementing | 2 (0-4) |
| Teso North | Comparison | 7 (1-14) |
| Teso South | Comparison | 6 (1-10) |
| **Homa Bay** |  |  |
| Homa Bay | Implementing | 5 (4-7) |
| Kabondo Kasipul | Implementing | 2 (1-4) |
| Karachuonyo | Comparison | 1 (0-4) |
| Kasipul | Comparison | 3 (0-6) |
| Ndhiwa | Implementing | 1 (0-2) |
| Rangwe | Comparison | 4 (0-9) |
| Mbita Suba North | Comparison | 5 (4-7) |
| Suba Suba South | Comparison | 12 (5-19) |
| **Kakamega** |  |  |
| Butere | Implementing | 8 (4-12) |
| Ikolomani | Implementing | 14 (4-24) |
| Khwisero | Implementing | 8 (3-13) |
| Lurambi | Comparison | 5 (0-12) |
| Malava | Implementing | 8 (6-9) |
| Matungu | Comparison | 15 (9-20) |
| Mumias East | Comparison | 9 (5-14) |
| Mumias West | Implementing | 10 (4-17) |
| Navakholo | Implementing | 8 (7-9) |
| Shinyalu | Comparison | 4 (3-5) |
| **Kisumu** |  |  |
| Kisumu Central | Implementing | 3 (0-7) |
| Kisumu East | Implementing | 7 (1-13) |
| Muhoroni | Implementing | 7 (3-11) |
| Nyakach | Comparison | 5 (2-8) |
| **Migori** |  |  |
| Awendo | Implementing | 7 (6-9) |
| Nyatike | Implementing | 2 (1-3) |
| Rongo | Comparison | 3 (0-6) |
| Suna East | Comparison | 9 (1-17) |
| Suna West | Comparison | 7 (4-9) |
| Uriri | Comparison | 4 (2-6) |
| **Siaya** |  |  |
| Rarieda | Implementing | 7 (5-8) |
| Ugenya | Implementing | 6 (2-10) |
| Ugunja | Comparison | 5 (4-6) |
| **Vihiga** |  |  |
| Emuhaya | Implementing | 6 (2-10) |
| Hamisi | Comparison | 3 (0-7) |
| Luanda | Implementing | 2 (1-4) |
| Sabatia | Comparison | 10 (6-13) |
| Vihiga | Implementing | 5 (1-9) |

**Table S5. Home-based record availability by subcounty**

| **Name** | **MVIP Allocation** | **HBR availability (for children 12-23 m.o.), %(95%CI)** |
| --- | --- | --- |
| **Bungoma** |  |  |
| Bumula | Implementing | 70 (45-96) |
| Kanduyi | Comparison | 90 (81-98) |
| Sirisia | Comparison | 87 (73-100) |
| **Busia** |  |  |
| Budalangi | Comparison | 85 (74-96) |
| Butula | Implementing | 98 (94-100) |
| Funyula | Implementing | 82 (66-99) |
| Matayos | Comparison | 94 (88-99) |
| Nambale | Implementing | 85 (73-97) |
| Teso North | Comparison | 83 (78-88) |
| Teso South | Comparison | 84 (65-100) |
| **Homa Bay** |  |  |
| Homa Bay | Implementing | 69 (49-90) |
| Kabondo Kasipul | Implementing | 85 (75-95) |
| Karachuonyo | Comparison | 79 (68-91) |
| Kasipul | Comparison | 82 (65-98) |
| Ndhiwa | Implementing | 94 (88-100) |
| Rangwe | Comparison | 75 (70-79) |
| Mbita Suba North | Comparison | 72 (67-78) |
| Suba Suba South | Comparison | 88 (85-91) |
| **Kakamega** |  |  |
| Butere | Implementing | 94 (86-100) |
| Ikolomani | Implementing | 75 (58-92) |
| Khwisero | Implementing | 84 (80-89) |
| Lurambi | Comparison | 88 (79-97) |
| Malava | Implementing | 93 (83-100) |
| Matungu | Comparison | 100 (100-100) |
| Mumias East | Comparison | 87 (75-99) |
| Mumias West | Implementing | 82 (74-90) |
| Navakholo | Implementing | 86 (76-97) |
| Shinyalu | Comparison | 96 (88-100) |
| **Kisumu** |  |  |
| Kisumu Central | Implementing | 100 (100-100) |
| Kisumu East | Implementing | 94 (89-99) |
| Muhoroni | Implementing | 84 (76-91) |
| Nyakach | Comparison | 86 (71-100) |
| **Migori** |  |  |
| Awendo | Implementing | 86 (79-93) |
| Nyatike | Implementing | 90 (85-95) |
| Rongo | Comparison | 93 (79-100) |
| Suna East | Comparison | 84 (76-92) |
| Suna West | Comparison | 74 (72-77) |
| Uriri | Comparison | 81 (72-91) |
| **Siaya** |  |  |
| Rarieda | Implementing | 81 (63-99) |
| Ugenya | Implementing | 91 (79-100) |
| Ugunja | Comparison | 97 (91-100) |
| **Vihiga** |  |  |
| Emuhaya | Implementing | 97 (93-100) |
| Hamisi | Comparison | 91 (79-100) |
| Luanda | Implementing | 88 (64-100) |
| Sabatia | Comparison | 100 (100-100) |
| Vihiga | Implementing | 88 (81-96) |
|  |  |  |

**Table S6. Availability of home-based vaccination records in children 5-48 months**

| **Background Characteristics** | **HBR availability** | |  | **Coverage ratio** |
| --- | --- | --- | --- | --- |
|  | **n unweighted** | **% (95% CI) weighted** |  | **%(95%CI)** |
| **Overall** | **3768/4948** | **76 (74, 77)** |  | **-** |
| **Age group** |  |  |  |  |
| 5-11 m.o. | 704/760 | 93 (91, 95) |  | Reference |
| 12-23 m.o. | 1141/1338 | 86 (83, 88) |  | 0.92 (0.89, 0.95)* |
| 24-35 m.o. | 492/681 | 69 (63, 74) |  | 0.74 (0.68, 0.80)* |
| *30-41 m.o.* | *453/636* | *71 (67, 75)* |  | *0.76 (0.71, 0.81)** |
| 36-48 m.o. | 978/1533 | 63 (60, 66) |  | 0.68 (0.64, 0.71)* |
| **Gender of child** |  |  |  |  |
| Female | 1831/2413 | 75 (73, 77) |  | 0.98 (0.95, 1.02) |
| Male | 1937/2535 | 76 (74, 78) |  | Reference |
| **Residence** |  |  |  |  |
| Urban | 786/1035 | 77 (73, 80) |  | Reference |
| Rural | 2982/3913 | 75 (73, 77) |  | 0.98 (0.93, 1.03) |
| **Randomization arm** |  |  |  |  |
| Implementing | 1860/2430 | 76 (74, 78) |  | 1.00 (0.96, 1.05) |
| Comparison | 1908/2518 | 75 (73, 78) |  | Reference |
| **Malaria prevalence** |  |  |  |  |
| Low | 1216/1672 | 73 (70, 77) |  | Reference |
| Medium | 1251/1617 | 76 (74, 79) |  | 1.05 (0.99, 1.11) |
| High | 1301/1659 | 77 (75, 80) |  | 1.06 (1.00, 1.12) |
| **Wealth Index** |  |  |  |  |
| Low | 1287/1678 | 77 (74, 79) |  | Reference |
| Medium | 1280/1706 | 74 (71, 77) |  | 0.97 (0.92, 1.02) |
| High | 1201/1564 | 76 (74, 79) |  | 1.00 (0.95, 1.04) |
| **IRS** |  |  |  |  |
| No IRS | 3142/4062 | 77 (75, 79) |  | Reference |
| IRS | 626/886 | 70 (65, 74) |  | 0.91 (0.85, 0.97)* |
| *p < 0.05 |  |  |  |  |

**Table S7a. Vaccination coverage (HBR only), by population characteristics**

| **HBR only** | **Vaccine** | **Dose** | **Background Characteristics** | | | | | | | |
| --- | --- | --- | --- | --- | --- | --- | --- | --- | --- | --- |
|  |  |  | **Overall** | | **Gender of child** | | **Residence** | | **Arm** | |
|  |  |  |  |  | ***Female*** | ***Male*** | ***Urban*** | ***Rural*** | ***Implementing*** | ***Comparison*** |
|  |  |  | n/N unweighted | % (95% CI) weighted | % (95% CI) weighted | % (95% CI) weighted | % (95% CI) weighted | % (95% CI) weighted | % (95% CI) weighted | % (95% CI) weighted |
|  | **BCG** | 1 | 1105/1141 | 95 (93, 98) | 97 (96, 99) | 94 (89, 99) | 91 (81, 100) | 96 (95, 98) | 95 (93, 98) | 95 (91, 100) |
|  | **DPT-HepB (Pentavalent)** | 1 | 1132/1141 | 99 (99, 100) | 1.00 (99, 100) | 99 (98, 100) | 100 ( ., .) | 99 (98, 100) | 99 (99, 100) | 99 (98, 100) |
|  |  | 2 | 1120/1141 | 98 (97, 99) | 99 (98, 1.00) | 97 (95, 98) | 100 ( ., .) | 97 (96, 98) | 98 (97, 99) | 98 (96, 99) |
|  |  | 3 | 1087/1141 | 95 (94, 96) | 96 (94, 98) | 94 (92, 96) | 98 (97, 100) | 94 (92, 96) | 94 (92, 97) | 95 (94, 97) |
|  | **Polio (OPV)** |  |  |  |  |  |  |  |  |  |
|  |  | 1 | 1134/1141 | 99 (98, 100) | 1.00 (99, .00) | 99 (97, 100) | 98 (95, 100) | 99 (99, 100) | 99 (99, 100) | 99 (97, 100) |
|  |  | 2 | 1123/1141 | 98 (97, 99) | 99 (98, 100) | 97 (95, 99) | 98 (95, 100) | 98 (97, 99) | 99 (98, 100) | 97 (96, 99) |
|  |  | 3 | 1070/1141 | 93 (91, 95) | 94 (92, 96) | 92 (88, 95) | 96 (93, 99) | 92 (90, 95) | 94 (90, 97) | 92 (90, 95) |
|  | **Pneumococcal (PCV)** | 1 | 1123/1141 | 99 (98, 99) | 99 (98, 100) | 98 (97, 99) | 1.00 (99, 100) | 98 (97, 99) | 99 (98, 100) | 98 (98, 99) |
|  |  | 2 | 1113/1141 | 97 (96, 98) | 98 (97, 100) | 96 (95, 98) | 99 (98, 100) | 97 (96, 98) | 97 (96, 99) | 97 (96, 99) |
|  |  | 3 | 1075/1141 | 94 (93, 96) | 96 (94, 98) | 93 (91, 95) | 96 (93, 99) | 94 (92, 96) | 93 (90, 95) | 96 (94, 97) |
|  | **Rotavirus** | 1 | 1124/1141 | 98 (97, 99) | 99 (98, 100) | 98 (96, 99) | 99 (98, 100) | 98 (97, 99) | 98 (96, 100) | 99 (98, 100) |
|  |  | 2 | 1087/1141 | 94 (93, 96) | 96 (94, 98) | 93 (91, 95) | 95 (92, 98) | 94 (92, 96) | 94 (92, 96) | 95 (93, 97) |
|  | **Measles** | 1 | 1020/1141 | 88 (86, 91) | 90 (88, 93) | 86 (83, 90) | 93 (89, 98) | 87 (84, 90) | 86 (82, 90) | 91 (87, 94) |
|  |  | 2 | 485/945 | 50 (46, 55) | 53 (47, 60) | 48 (43, 53) | 55 (46, 66) | 49 (44, 54) | 49 (44, 54) | 52 (45, 59) |
|  | **Fully vaccinated** | (ba  sic) | 914/1141 | 79 (76, 82) | 82 (78, 85) | 76 (71, 82) | 80 (73, 88) | 78 (75, 82) | 76 (72, 81) | 81 (77, 86) |

**Table S7b. Vaccination coverage (HBR only), by population characteristics (continued)**

| **HBR only** | **Vaccine** | **Dose** | **Background Characteristics** | | | | | | | |
| --- | --- | --- | --- | --- | --- | --- | --- | --- | --- | --- |
|  |  |  | **Malaria Prevalence** | | | **Wealth** | | | **IRS** | |
|  |  |  | ***Low*** | ***Middle*** | ***High*** | ***Low*** | ***Middle*** | ***High*** | ***No IRS*** | ***IRS*** |
|  |  |  | % (95% CI) weighted | % (95% CI) weighted | % (95% CI) weighted | % (95% CI) weighted | % (95% CI) weighted | % (95% CI) weighted | % (95% CI) weighted | % (95% CI) weighted |
|  | **BCG** | 1 | 94 (87, 100) | 97 (95, 99) | 95 (91, 98) | 97 (96, 99) | 95 (91, 99) | 94 (90, 97) | 95 (92, 98) | 98 (95, 100) |
|  | **DPT-HepB (Pentavalent)** | 1 | 99 (98, 100) | 99 (98, 100) | 99 (99, 100) | 99 (98, 100) | 99 (98, 100) | 100 (99, 100) | 99 (99, 100) | 98 (96, 100) |
|  |  | 2 | 99 (98, 100) | 97 (95, 99) | 98 (97, 99) | 97 (95, 99) | 98 (97, 100) | 99 (98, 100) | 98 (97, 99) | 95 (91, 100) |
|  |  | 3 | 96 (94, 99) | 93 (90, 96) | 96 (94, 97) | 93 (91, 96) | 96 (93, 98) | 96 (93, 98) | 96 (95, 97)* | 90 (85, 95)* |
|  | **Polio (OPV)** | 0 |  |  |  |  |  |  |  |  |
|  |  | 1 | 98 (96, 100) | 99 (98, 100) | 100 (99, 100) | 100 (99, 100) | 98 (96, 100) | 100 (99, 100) | 99 (98, 100) | 98 (96, 100) |
|  |  | 2 | 98 (96, 100) | 97 (96, 99) | 99 (98, 100) | 99 (97, 100) | 97 (94, 99) | 99 (98, 100) | 98 (97, 99) | 97 (95, 100) |
|  |  | 3 | 95 (92, 97) | 90 (85, 95) | 95 (93, 98) | 92 (86, 98) | 93 (89, 96) | 95 (92, 98) | 94 (92, 96) | 90 (85, 95) |
|  | **Pneumococcal (PCV)** | 1 | 99 (98, 100) | 99 (98, 100) | 98 (97, 99) | 98 (96, 99) | 98 (97, 100) | 99 (99, 100) | 99 (98, 99) | 98 (95, 100) |
|  |  | 2 | 99 (98, 100) | 96 (94, 98) | 97 (96, 99) | 96 (94, 99) | 97 (96, 99) | 98 (97, 100) | 98 (97, 99) | 95 (91, 99) |
|  |  | 3 | 96 (93, 98) | 93 (90, 96) | 95 (93, 97) | 93 (91, 96) | 94 (91, 97) | 96 (93, 98) | 95 (94, 97) | 90 (85, 95) |
|  | **Rotavirus** | 1 | 99 (98, 100) | 97 (95, 99) | 99 (98, 100) | 98 (96, 100) | 98 (96, 100) | 99 (97, 100) | 98 (97, 99) | 97 (94, 100) |
|  |  | 2 | 95 (93, 98) | 92 (89, 96) | 96 (94, 98) | 94 (91, 97) | 95 (93, 97) | 94 (92, 97) | 95 (93, 96) | 92 (88, 97) |
|  | **Measles** | 1 | 89 (85, 94) | 84 (79, 90) | 92 (88, 96) | 82 (76, 88)* | 90 (87, 93)* | 92 (89, 96)* | 90 (87, 93)* | 80 (73, 88)* |
|  |  | 2 | 54 (47, 62) | 45 (39, 53) | 53 (46, 60) | 42 (35, 51)* | 52 (46, 59)* | 55 (48, 62)* | 53 (49, 59)* | 36 (28, 47)* |
|  | **Fully vaccinated** | (ba  sic) | 81 (75, 87) | 77 (71, 82) | 79 (75, 84) | 74 (68, 80) | 81 (75, 86) | 81 (78, 86) | 79 (76, 83) | 75 (68, 83) |

Note: vaccine coverage is measured in children 12-23 months old for all vaccinations in first year of life; measles dose 2 coverage is measured in children 24-35 months of age. * p<0.05 based on prevalence ratios

**Table S8. Vaccination coverage by different indicators (HBR alone and HBR or recall, with two different denominator treatments)**

|  | **(I) HBR only** (denominator excludes missing HBR) | **(II) HBR only** (denominator includes missing coded as "not vaccinated") | **(III) HBR and recall** (denominator excludes missing HBR and recall) | **(IV) HBR and recall** (denominator includes missing coded as "not vaccinated") |
| --- | --- | --- | --- | --- |
| **Vaccines** | **% (95%CI)** | **% (95%CI)** | **% (95%CI)** | **% (95%CI)** |
| **BCG** | 99 (98-99) | 82 (79-85) | 99 (98-100) | 99 (98-99) |
| **OPV1** | 97 (96-98) | 85 (83-87) | 99 (98-99) | 97 (96-98) |
| **OPV2** | 95 (94-97) | 84 (82-87) | 97 (96-98) | 95 (94-97) |
| **OPV3** | 91 (89-93) | 83 (31-86) | 93 (91-95) | 91 (89-93) |
| **Penta1** | 97 (96-98) | 85 (83-88) | 98 (97-99) | 97 (96-98) |
| **Penta2** | 96 (94-97) | 84 (81-86) | 97 (96-98) | 96 (94-97) |
| **Penta3** | 91 (88-93) | 81 (79-84) | 92 (89-94) | 91 (88-93) |
| **PCV 1** | 95 (93-96) | 84 (86-87) | 96 (95-97) | 95 (93-96) |
| **PCV 2** | 92 (90-94) | 83 (81-86) | 93 (92-95) | 92 (90-94) |
| **PCV 3** | 88 (86-90) | 81 (78-84) | 89 (87-92) | 88 (86-90) |
| **Rota 1** | 94 (92-95) | 84 (82-87) | 96 (94-97) | 94 (92-95) |
| **Rota 2** | 88 (86-90) | 81 (78-84) | 90 (88-92) | 88 (86-90) |
| **Measles-1** | 89 (87-92) | 76 (72-79) | 90 (88-93) | 89 (97-92) |
| **Measles-2** | 50 (45-55) | 37 (32-42) | 51 (46-57) | 57 (53-62) |

Note: vaccine coverage is measured in children 12-23 months old for all vaccinations in first year of life; measles dose 2 coverage is measured in children 24-35 months of age.

**Table S9. Vaccination coverage (HBR only), by county**

|  | **County** | | | | | | | |
| --- | --- | --- | --- | --- | --- | --- | --- | --- |
| **County** | **Bungoma** | **Busia** | **Homa Bay** | **Kakamega** | **Kisumu** | **Migori** | **Siaya** | **Vihiga** |
|  | **% (95%CI)** | **% (95%CI)** | **% (95%CI)** | **% (95%CI)** | **% (95%CI)** | **% (95%CI)** | **% (95%CI)** | **% (95%CI)** |
| **Vaccination-related indicators (children 12-23 m.o.)** | |  |  |  |  |  |  |  |
| **Children with home-based vaccination record available** | 82 (72 - 93) | 88 (82 - 93) | 82 (76 - 87) | 89 (85 - 93) | 91 (85 - 96) | 84 (80 - 89) | 87 (77 - 97) | 93 (88 - 98) |
| **BCG coverage** | 93 (88 - 99) | 97 (95 - 99) | 97 (94 - 99) | 89 (81 - 97) | 97 (93 - 100) | 99 (97 - 100) | 100 (100 - 100) | 94 (89 - 99) |
| **OPV Birth coverage** | 99 (97 - 100) | 99 (98 - 100) | 97 (94 - 99) | 99 (96 - 100) | 97 (93 - 100) | 100 (99 - 100) | 100 (100 - 100) | 99 (97 - 100) |
| **OPV 1 coverage** | 99 (97 - 100) | 99 (97 - 100) | 95 (92 - 98) | 98 (95 - 100) | 95 (91 - 99) | 99 (98 - 100) | 100 (100 - 100) | 99 (97 - 100) |
| **OPV 2 coverage** | 98 (95 - 100) | 96 (92 - 100) | 94 (90 - 97) | 97 (95 - 99) | 95 (91 - 99) | 98 (96 - 100) | 100 (100 - 100) | 98 (96 - 100) |
| **OPV 3 coverage** | 72 (65 - 79) | 73 (68 - 79) | 73 (62 - 84) | 78 (71 - 85) | 82 (76 - 89) | 77 (69 - 85) | 91 (86 - 97) | 89 (82 - 95) |
| **Penta 1 coverage** | 99 (97 - 100) | 98 (96 - 100) | 97 (94 - 99) | 100 (99 - 100) | 96 (91 - 100) | 99 (98 - 100) | 100 (100 - 100) | 99 (97 - 100) |
| **Penta 2 coverage** | 98 (95 - 100) | 96 (93 - 98) | 94 (90 - 97) | 99 (98 - 100) | 95 (91 - 99) | 98 (96 - 100) | 100 (100 - 100) | 98 (96 - 100) |
| **Penta 3 coverage** | 96 (93 - 99) | 95 (92 - 98) | 89 (83 - 95) | 96 (94 - 99) | 93 (88 - 98) | 93 (89 - 97) | 98 (94 - 100) | 97 (94 - 100) |
| **PCV 1 coverage** | 99 (97 - 100) | 94 (91 - 98) | 97 (94 - 99) | 100 (99 - 100) | 95 (91 - 99) | 98 (96 - 100) | 100 (100 - 100) | 99 (97 - 100) |
| **PCV 2 coverage** | 98 (95 - 100) | 94 (91 - 98) | 94 (90 - 97) | 99 (97 - 100) | 95 (91 - 99) | 97 (95 - 100) | 100 (100 - 100) | 97 (94 - 100) |
| **PCV 3 coverage** | 97 (93 - 100) | 93 (89 - 96) | 89 (83 - 95) | 96 (93 - 98) | 93 (88 - 98) | 92 (87 - 96) | 96 (92 - 100) | 95 (91 - 100) |
| **Rota 1 coverage** | 99 (97 - 100) | 97 (94 - 99) | 97 (94 - 99) | 98 (97 - 100) | 96 (91 - 100) | 96 (91 - 100) | 99 (96 - 100) | 99 (97 - 100) |
| **Rota 2 coverage** | 94 (91 - 97) | 93 (89 - 97) | 90 (85 - 95) | 94 (91 - 97) | 93 (88 - 98) | 94 (89 - 99) | 97 (92 - 100) | 96 (93 - 99) |
| **Measles 1 coverage** | 83 (74 - 92) | 93 (88 - 99) | 78 (67 - 89) | 96 (93 - 99) | 84 (77 - 91) | 78 (71 - 86) | 94 (90 - 98) | 94 (89 - 100) |
| **Measles 2 coverage (24-35m)** | 35 (23 - 47) | 55 (37 - 72) | 36 (20 - 52) | 52 (40 - 65) | 36 (23 - 49) | 29 (9 - 49) | 60 (45 - 75) | 58 (40 - 75) |

**Table S10. Vitamin supplementation in children 5-11 months and deworming, by population characteristics**

| **Background Characteristics** | **Vitamin A supplementation in the past 6 months, HBR or recall** | | | **Deworming, by recall** | | |
| --- | --- | --- | --- | --- | --- | --- |
|  | **n/N, unweighted** | **% (95% CI) weighted** | **PR (95%CI)** | **n/N, unweighted** | **% (95% CI) weighted** | **PR(95%CI)** |
| **Overall** | **230/506** | **46 (41,52)** | **-** | **245/494** | **50 (45,55)** | **-** |
| **Sex** |  |  |  |  |  |  |
| Female | 111/230 | 51 (44,59) | 1.22 (0.99,1.51) | 117/225 | 51 (44,59) | 1.06 (0.89,1.26) |
| Male | 119/276 | 42 (35,50) | Reference | 128/269 | 48 (43,55) | reference |
| **Residence** |  |  |  |  |  |  |
| Urban | 60/96 | 64 (55,74) | Reference | 52/95 | 57 (50,65) | reference |
| Rural | 170/410 | 42 (37,47) | 0.66 (0.54,0.80)* | 193/399 | 48 (42,54) | 0.84 (0.69,1.02) |
| **Randomization arm** | |  |  |  |  |  |
| Implementing | 124/251 | 48 (41,56) | 1.07 (0.83,1.38) | 115/244 | 45 (39,52) | 0.83 (0.68,1.02) |
| Comparison | 106/255 | 45 (36,55) | Reference | 130/250 | 54 (47,62) | Reference |
| **Malaria prevalence** | | | | | | |
| Low | 74/150 | 52 (41,65) | Reference | 79/147 | 57 (51,64) | Reference |
| Medium | 75/175 | 40 (34,49) | 0.78 (0.58,1.05) | 82/169 | 44 (35,57) | 0.78 (0.59,1.02) |
| High | 81/181 | 47 (39,56) | 0.91 (0.67,1.22) | 84/178 | 47 (41,55) | 0.83 (0.69, 1.00) |
| **Wealth Index** |  |  |  |  |  |  |
| Low | 61/162 | 37 (30,46) | Reference | 60/157 | 37 (30,45) | Reference |
| Medium | 76/176 | 45 (36,56) | 1.23 (0.94,1.62) | 79/173 | 44 (36,55) | 1.21 (0.90,1.62) |
| High | 93/168 | 55 (48,63) | 1.51 (1.17,1.94)* | 106/164 | 66 (59,74) | 1.80 (1.43,2.27)* |
| **IRS area** |  |  |  |  |  |  |
| No IRS | 200/437 | 47 (41,54) | Reference | 211/427 | 50 (44,56) | Reference |
| IRS | 30/69 | 41 (31,54) | 0.88 (0.66,1.18) | 34/67 | 49 (38,63) | 0.98 (0.74,1.29) |
| p<0.05 |  |  |  |  |  |  |

**Table S11. Measures of agreement in vaccinations reporting between HBR and caregiver recall, among children aged 12-23 months old with available HBR**

| **Vaccine** | **Coverage by HBR** | **Coverage by recall** | **Percent agreement** | **AC1 agreement coefficient (95% CI)** | **Brennan-Prediger coefficient (95% CI)** |
| --- | --- | --- | --- | --- | --- |
| **BCG** | **96%** | **98%** | **95%** | **0.94 (0.93,0.96)** | **0.92 (0.90,0.94)** |
| **OPV (any doses)** | **100%** | **99%** | **97%** | **0.97 (0.96,0.98)** | **0.96 (0.94,0.97)** |
| OPV1 | 98% | 88% | 86% | 0.85 (0.81,0.88) |  |
| OPV2 | 96% | 61% | 62% | 0.45 (0.40,0.51) |  |
| OPV3 | 79% | 78% | 65% | 0.56 (0.53,0.60) |  |
| **PCV (any doses)** | **98%** | **92%** | **79%** | **0.77 (0.75,0.80)** | **0.69 (0.66,0.73)** |
| PCV1 | 97% | 79% | 78% | 0.72 (0.68,0.76) |  |
| PCV2 | 96% | 70% | 69% | 0.57 (0.53,0.62) |  |
| PCV3 | 93% | 54% | 55% | 0.29 (0.23,0.35) |  |
| **Penta (any doses)** | **99%** | **99%** | **95%** | **0.94 (0.93,0.96)** | **0.92 (0.90,0.94)** |
| Penta1 | 98% | 95% | 93% | 0.93 (0.91,0.95) |  |
| Penta2 | 97% | 90% | 89% | 0.88 (0.85,0.90) |  |
| Penta3 | 94% | 72% | 73% | 0.63 (0.56,0.67) |  |
| **Rota (any doses)** | **98%** | **89%** | **78%** | **0.76 (0.73,0.79)** | **0.67 (0.64,0.71)** |
| Rota1 | 97% | 77% | 77% | 0.70 (0.66,0.74) |  |
| Rota2 | 94% | 53% | 55% | 0.28 (0.22,0.34) |  |
| **Measles (any doses)** | **90%** | **91%** | **90%** | **0.89 (0.87,0.91)** | **0.85 (0.82,0.87)** |
| MSL1 | 89% | 87% | 90% | 0.87 (0.85,0.90) |  |
| MSL2 | 47% | 46% | 78% | 0.57 (0.50,0.65) |  |
| **Vitamin A** | **80%** | **84%** | **78%** | **0.74 (0.71,0.77)** | **0.67 (0.63,0.70)** |
